# Somatic mutations in early onset luminal breast cancer

**DOI:** 10.18632/oncotarget.25123

**Published:** 2018-04-27

**Authors:** Giselly Encinas, Veronica Y. Sabelnykova, Eduardo Carneiro de Lyra, Maria Lucia Hirata Katayama, Simone Maistro, Pedro Wilson Mompean de Vasconcellos Valle, Gláucia Fernanda de Lima Pereira, Lívia Munhoz Rodrigues, Pedro Adolpho de Menezes Pacheco Serio, Ana Carolina Ribeiro Chaves de Gouvêa, Felipe Correa Geyer, Ricardo Alves Basso, Fátima Solange Pasini, Maria del Pilar Esteves Diz, Maria Mitzi Brentani, João Carlos Guedes Sampaio Góes, Roger Chammas, Paul C. Boutros, Maria Aparecida Azevedo Koike Folgueira

**Affiliations:** ^1^ Instituto do Cancer do Estado de Sao Paulo, Departamento de Radiologia e Oncologia, Faculdade de Medicina FMUSP, Universidade de Sao Paulo, Sao Paulo, SP, Brazil; ^2^ Ontario Institute for Cancer Research, Toronto, Canada; ^3^ Instituto Brasileiro de Controle do Câncer, São Paulo, Brazil; ^4^ Department of Medical Biophysics, University of Toronto, Toronto, Canada; ^5^ Department of Pharmacology and Toxicology, University of Toronto, Toronto, Canada

**Keywords:** breast cancer, young patients, somatic mutation, germline mutation, luminal subtype

## Abstract

Breast cancer arising in very young patients may be biologically distinct; however, these tumors have been less well studied. We characterized a group of very young patients (≤ 35 years) for BRCA germline mutation and for somatic mutations in luminal (*HER2* negative) breast cancer. Thirteen of 79 unselected very young patients were *BRCA*1/2 germline mutation carriers. Of the non-*BRCA* tumors, eight with luminal subtype (*HER2* negative) were submitted for whole exome sequencing and integrated with 29 luminal samples from the COSMIC database or previous literature for analysis. We identified C to T single nucleotide variants (SNVs) as the most common base-change. A median of six candidate driver genes was mutated by SNVs in each sample and the most frequently mutated genes were *PIK3CA, GATA3, TP53* and *MAP2K4*. Potential cancer drivers affected in the present non-*BRCA* tumors include *GRHL2, PIK3AP1, CACNA1E*, *SEMA6D*, *SMURF2*, *RSBN1* and *MTHFD2.* Sixteen out of 37 luminal tumors (43%) harbored SNVs in DNA repair genes, such as *ATR, BAP1, ERCC6, FANCD2, FANCL, MLH1*, *MUTYH, PALB2, POLD1, POLE*, *RAD9A, RAD51 and TP53*, and 54% presented pathogenic mutations (frameshift or nonsense) in at least one gene involved in gene transcription. The differential biology of luminal early-age onset breast cancer needs a deeper genomic investigation.

## INTRODUCTION

Breast cancer mainly affects post-menopausal women, however, it is estimated that 4.8-5.0% of cases occur in young adults, less than 40 years [1]. Even at this early age the disease can be highly fatal. In the USA, where cancer is the second leading cause of total deaths in young women aged less than 40 years, breast cancer is the leading cause of cancer deaths in this age group [2].

There is evidence that some cancers in very young adults have differential biology, and probably etiology/pathogenesis, compared to older persons [3]. Surprisingly, only a few studies have explored this question. In breast cancer, germline mutations in *BRCA1* and *BRCA2* genes may support the carcinogenic process in around 20% of the young patients [4–7], but only in 1-4% of post-menopausal women [8]. Mutations in other cancer predisposing genes such as *TP53*, *PTEN, CHEK2*, may explain an additional 4% of early onset cases [9]

Younger age has been associated with a less favorable prognosis in breast cancer partly because early onset cases comprise a lower proportion of the relatively good outcome luminal A subtype and higher proportion of the more aggressive triple negative subtype. Moreover, within each subtype, women diagnosed at an early age may have worse outcomes than those diagnosed at more advanced ages in any breast cancer subtype, i.e., luminal [10–12], triple negative and HER2 [13]. Although young age seems to be a poor prognostic factor, different age cut offs have been used, varying from 35 [10, 13] to 40 years [11, 12]. It is interesting to observe that women aged less than 35 years old seems to have similar disease-free survival among themselves, which is worse compared with women aged 35 to 50 years old [10, 13].

Accordingly, mRNA abundance analysis revealed a differential transcriptional profile in tumors arising in young women, with enrichment of biological processes related to immature mammary cell populations and growth factor signaling [11]. Molecular signatures of breast cancer subtypes, irrespective of age, have been examined and great differences have been shown between basal-like and luminal tumors, the former presenting a higher rate of genomic rearrangements than the latter [14]. In addition, numerous subtype-associated and novel gene mutations have been described [15–18]. These studies however, have not focused on somatic point mutations (single nucleotide variants, SNVs) that may distinguish early onset breast cancer. Hence, our aims were to characterize a group of Brazilian patients with early onset breast cancer for *BRCA1* and *BRCA2* germline mutations, as well as for somatic SNVs arising in luminal subtype tumors.

## RESULTS

### Family history suggestive of hereditary breast and ovarian cancer syndrome (HBOCS) and germline *BRCA1* and *BRCA2* mutations

Our first aim was to evaluate family history of cancer and to detect *BRCA1* and *BRCA2* mutations in very young Brazilian patients. For this purpose, 79 young women were interviewed, among whom 17 (21.5%) were not able to provide family history for one or both sides of the family. Thirty (48.3%) out of 62 patients with informative family history reported at least one close relative (until 3^rd^ degree) with breast, ovarian, pancreatic or prostate cancer, among whom 10 (16.2%) reported at least one affected first degree family member (Supplementary Table 1).

Thirteen out of 79 patients presented pathogenic mutations (16.5%) in *BRCA1* or *BRCA2* genes. These, represent 12 distinct types of mutations: three frameshift and one missense in *BRCA1* and four frameshift, three nonsense and one missense in *BRCA2*. Only one mutation (frameshift mutation in *BRCA2* c.2808_2811delACAA (p.Ala938Profs) was detected in two women; one nonsense mutation c.483T>A (C161X) on exon 6 of *BRCA2* was detected for the first time (Supplementary Tables 2 and 3).

Twenty-nine variants of uncertain significance (VUS) were also identified, including 13 distinct missense variants, each one detected only once: two in *BRCA1* and 11 in *BRCA2* gene. Two patients presented more than one (missense) VUS in *BRCA2* gene, one of them, diagnosed with a triple negative tumor reported a positive family history (c.3349A>G; c.5414A>G; c.8092G>A); the other one, diagnosed with a luminal B tumor, presented a limited family history (c.2837A>G; c.7418G>A). In addition, one VUS characterized as an in-frame deletion in exon 23 of *BRCA1* gene (c.5425_5430delGTTGTG) (Supplementary Table 4), was observed in a patient with positive family history, diagnosed with triple negative breast cancer. The other VUS were characterized as intronic or synonymous variants.

Neither of these patients presented large deletions/amplifications in *BRCA1* and *BRCA2*, nor *CHEK2* mutations (c.1100delC).

### Somatic SNVs detected by whole exome sequencing

Eight patients, who were *BRCA1* and *BRCA2* wild type carriers with luminal HER2 negative tumors, had their tumor and normal exomes sequenced. These patients mainly reported Brazilian ancestry in both sides of the family, which means that their parents and grandparents were born in Brazil, but they were not aware from where did more ancient ancestries had come from. One patient reported one maternal grandmother with Amerindian ancestry and a second patient reported grandparents from the paternal side with European ancestry.

**Figure 1:**
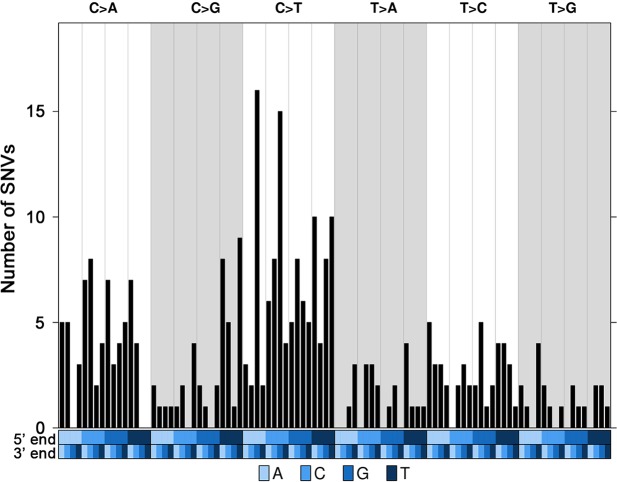
Trinucleotide mutational profile of current luminal samples Trinucleotide barplot showing the number of Single Nucleotide Variants (SNVs) in the context of each of the 96 trinucleotide mutation types. The blue covariates at the bottom of the plot represent the 5' and 3' ends. All the 310 SNVs were considered.

Whole exome sequencing of these eight tumors and matched blood samples was performed to a mean sequencing depth of 35.8x for tumors and 36.3x for corresponding blood samples (Supplementary Table 5). The mean total mutation rate across all samples was 1.9/Mbp. The mean non-silent mutation rate was 1.8/Mbp (Supplementary Table 6) and the most frequent events were C to T transitions, mainly seen in trinucleotides ACG>ATG and CCT>CTG (Figure 1).

We identified 310 somatic single nucleotide variants (SNVs), comprising of 303 unique variants (five SNVs were detected in two patients each; one SNV was detected in three patients), and mainly comprising intergenic regions, 3 prime UTR, missense, intron and synonymous variants (Supplementary Table 7). The median mutation load was 37.5 and varied from 19-74 SNVs per tumor.

**Figure 2:**
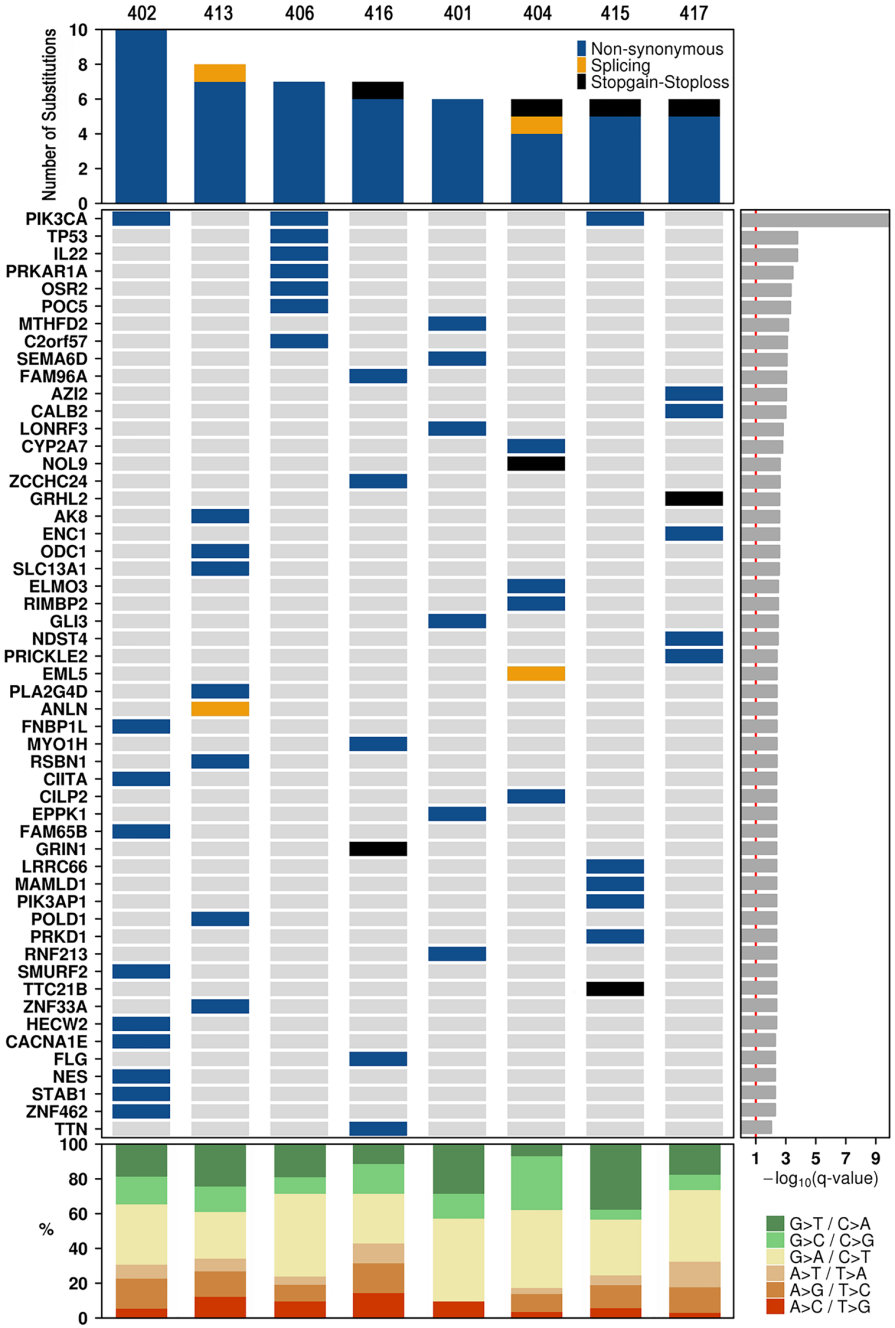
Landscape of coding somatic SNVs Each of the 54 genes in which at least one significant SNV was identified is listed down the left hand side. The genes are listed by their significant SeqSig q-value (FDR adjusted p-value). Type and number of mutations (top panel), significantly mutated genes (middle panel) and percentage of Single Nucleotide Variants (SNVs) (bottom panel) per tumor sample.

SeqSig analysis revealed 55 likely driver non-synonymous mutations in 53 genes (false discovery rate, (FDR) < 10%); (Figure 2) and *PIK3CA* was the only recurrent finding, which was detected in three different tumors. Somatic SNVs were then verified by performing an independent capillary sequencing (except for *GLI3, LONRF3 and EPPK1* that were not tested) and 81% (42/52) were confirmed (Supplementary Tables 8, 8a). Confirmed SNVs included nonsense mutations in four genes, *GRHL2*, *GRIN1*, *NOL9* and *TTC21B*, as well as 38 missense mutations in 36 different genes, including known tumor suppressor genes, such as *TP53* and *POLD1*, and protein kinases like *PRKD1*, *PRKAR1A* and *AK8*.

We compared our results with the gene list from the “Cancer Gene Census” database (http://cancer.sanger.ac.uk/census) [19] and detected five genes, *PIK3CA, TP53, PRKAR1A, POLD1 and CIITA*, which were already causally implicated in cancer.

We have then examined more closely this list of candidates to identify potentially cancer driver genes, using the score system described in methods, mainly based on detection in databases of mouse insertional mutagenesis experiments and causal relationship mutation function assessment algorithms, Kaplan-Meier (KM) plotter [20] (to assess the effect of the genes on breast cancer prognosis) (Supplementary Figure 1) and literature, among others.

Excluding the five genes included in the “Cancer Census Gene” database, another 18 genes were already reported as candidate cancer genes through transposon-based forward genetic screens in mice [21] (Table 1). Seven genes were relatively frequently mutated (≥1%) in cancer in general or in breast cancer specifically: *CACNA1E, HECW2, STAB1, ZNF462, FLG, TTN* and *NDST4.* Finally, using the above ranking system, 20 genes, scoring at least 2, were considered possible cancer drivers (Supplementary Tables 9, 9a), such as *PIK3AP1*, *GRHL2*, *CACNA1E*, *SMURF2, SEMA6D, RSBN1, MTHFD2*, among others.

**Table 1:** Cancer-related analysis of confirmed gene variants detected in breast cancer samples in the current analysis

ID	Gene	Alteration	CGC	CCGD (mice)	Mutation domain	Same variant in BC/Other Cancers	SNVs Frequency in all cancers	SNVs Frequency in BC	SNVs in BC young patients/all ages	FATHMM (score)	PolyPhen	SIFT	GV/GD	CRAVAT - CHASM p-value (missense)	KM - OS	Literature	Score	Total
401	*MTHFD2*	p.P17L c.50C>T	No	Blood - D [1], Colorectal - NR [2]	low_complexitySource: segmasker	No/No	38/37401 (0.10%)	1/2137 (0.05%)	0/1	Pathogenic (0.87)	Benign	Tolerated	0.00/97.78 (C65)	0.2851	p = 0.0027 OE	[3–5]	2.5	pd
401	*SEMA6D*	p.E553A c.1658A>C	No	Sarcoma - B [6] Colorectal - B [7]	Plexin Repeat	No/No	317/37626 (0.84%)	17/2159 (0.78%) 1 FS	2/17	Pathogenic (0.74)	ND	Tolerated	0.00/106.71 (C65)	0.3967	p = 0.0091 n≤200	[8]	3	pd
402	*CACNA1E*	p.R590W c.1768C>T	No	No results	Ion transport Domain	No/Yes	902/37516 (2.40%)	54/2116 (2.55%) 2 NS	3/45	Pathogenic (0.89)	Deleterious	Not Tolerated	0.00/101.29 (C65)	0.0523	p = 0.11 n≤200	[9, 10]	3	pd
402	*CIITA*	p.P443T c.1327C>A	Yes	No results	NACHT domain	No/No	2/37750 (0.005%)	0	-	Pathogenic (0.58)	Deleterious	Tolerated	0.00/37.56 (C35)	0.3563	p = 0.085	[11–13]	4.5	CGC
402	*FAM65B/RIPOR2*	p.E718D c.2154G>C	No	Blood - C [14]	No Pfam annotations found	No/No	130/37401 (0.35%)	7/2114 (0.33%) 1 FS	1/7	Neutral (0.26)	ND	Tolerated	0.00/44.60 (C35)	0.8042	p = 0.14	NO	1	Neutral
402	*HECW2*	p.D265G c.794A>G	No	Liver - D [15]	C2 Domain	No/No	422/38016 (1.11%)	20/2312 (0.86%)	1/20	Pathogenic (0.98)	Deleterious	Not Tolerated	0.00/93.77 (C65)	0.058	p = 0.42 n≤200	[16]	3	pd
402	*NES*	p.E340V c.1019A>T	No	No results	No fuctional domain	No/No	346/37419 (0.92%)	17/2137 (0.79%) 1 NS	0/17	Pathogenic (0.59)	Deleterious	Not Tolerated	0.00/121.33 (C65)	0.1896	p = 0.028 UE	[17–21]	3	pd
402	*PIK3CA*	p.E545K c.1633G>A	Yes	Blood - D [1] Gastric - D [22] Liver - C [23, 24] Nervous system - D [25] Skin - B [26]	PIK domain	Yes/Yes	10271/107457 (9.56%)	4098/15384 (26.64%)	-	Pathogenic (0.97)	Deleterious	Tolerated	0.00/56.87 (C55)	0.0002	p = 0.057	Oncogene	7.5	CGC
402	*SMURF2*	p.S193C c.578C>G	No	Blood - A [1] Colorectal - C [2, 27] Gastric - C [22] Liver - A [15, 23] Pancreatic – D [28]	disorderSource: IUPred	No/No	117/38086 (0.30%)	9/2288 (0.39) 3 FS	1/9	Pathogenic (0.91)	Benign	Not Tolerated	0.00/111.67 (C65)	0.4405	p = 0.074 n≤200	[29, 30]	4	PD
402	*STAB1*	p.G1381R c.4141G>A	No	No results	No fuctional domain	No/No	502/37566 (1.34%)	23/2158 (1.06%) 3 FS/2 NS	0/22	Pathogenic (0.72)	Deleterious	Tolerated	0.00/125.13 (C65)	0.6908	p = 0.071	[31–33]	2.5	pd
402	*ZNF462*	p.G2426C c.7276G>T	No	Breast - C [34]	No fuctional domain	No/No	555/37476 (1.48%)	36/2137 (1.68%) 2 NS	3/36	Pathogenic (0.89)	Deleterious	Tolerated	0.00/158.23 (C65)	0.0004	p = 0.028 n≤200	NO	2.5	pd
404	*CILP2*	p.R472G c.1414C>G	No	Blood - D [1]	No fuctional domain	No/No	309/37401 (0.83%)	5/2137 (0.23%) 1 NS	0/5	Neutral (0.1)	Deleterious	Not Tolerated	0.00/125.13 (C65)	0.661	p = 0.17 n≤200	NO	1.5	Neutral
404	*ELMO3*	p.L251F c.753G>C	No	No results	No Pfam annotations found	No/No	117/37401 (0.31%)	2/2137 (0.09%)	1/2	Pathogenic (0.66)	Benign	Tolerated	0.00/21.82 (C15)	0.0675	p = 0.000052 OE	[35–37]	2	pd
404	*NOL9*	p.S283^*^ c.848C>G	No	No results	low_complexitySource: segmasker	No/No	111/37401 (0.30%)	5/2137 (0.23%) 1 FS	1/5	Neutral (0.13)	ND	ND	-	-	p = 0.15	NO	1.5	Neutral
406	*C2orf57/TEX44*	p.T265M c.794C>T	No	Blood - D [1]	Domain of unknown function	No/Yes	89/37401 (0.24%)	1/2137 (0.05%) 1 NS	0/1	Neutral (0.00)	Benign	Tolerated	0.00/81.04 (C65)	0.5572	p = 0.14 n≤200	NO	0.5	Neutral
406	*IL22*	p.R73C c.217C>T	No	No results	Interleukin 22 domain	No/No	60/37402 (0.16%)	3/2137 (0.14%)	0/3	Pathogenic (0.57)	Deleterious	Not Tolerated	0.00/179.53 (C65)	0.6159	p = 0.4 n≤200	[38–40]	2.5	pd
406	*OSR2*	p.G262E c.785G>A	No	No results	Zinc Finger domain	No/No	98/37312 (0.26%)	5/2126 (0.24%) 1 FS	0/5	Pathogenic (0.94)	Deleterious	Not Tolerated	0.00/97.85 (C65)	0.0922	p = 0.2	[41]	2.5	pd
406	*PIK3CA*	p.H1047R c.3140A>G	Yes	Blood - D [1] Gastric - D [22] Liver - C [23, 24] Nervous system - D [25] Skin - B [26]	PI3K/PI4K domain	Yes/Yes	10271/107457 (9.56%)	4098/15384 (26.64%)	-	Pathogenic (0.96)	Deleterious	Tolerated	0.00/28.82 (C25)	0	p = 0.057	Oncogene	7.5	CGC
406	*POC5*	p.R541Q c.1622G>A	No	No results	No Pfam annotations found	No/No	87/37355 (0.23%)	3/2137 (0.14%) 1 FS	2/3	Pathogenic (0.94)	Deleterious	Not Tolerated	0.00/48.81 (C35)	0.4111	p = 0.06 n≤200	NO	1	Neutral
406	*PRKAR1A*	p.L20F c.58C>T	Yes	Liver - B [15, 23] Nervous system - D [25]	Dimerization and phosphorylation region	No/No	112/40450 (0.28%)	8/2379 (0.34%) 2 NS	0/8	Pathogenic (0.94)	Benign	Tolerated	0.00/21.82 (C15)	0.3232	p = 0.14	[42–44]	6	CGC
406	*TP53*	p.T220C c.659A>G	Yes	Colorectal - C [2, 27, 45] Nervous system - NR [46] Skin- A [47] Liver - NR [24]	P53 DNA-binding domain	Yes/Yes	31140/127779 (24.37%)	3189/13359 (23.87%)	-	Pathogenic (0.99)	Deleterious	Not Tolerated	-	0.0012	p = 0.041 UE	TSG	9	CGC
413	*AK8*	p.T101P c.301A>C	No	Blood - B [1]	Adenylate kinase	No/No	106/37402 (0.28%)	3/2114 (0.14%)	1/3	Neutral (0.02)	Benign	Tolerated	0.00/37.56 (C35)	0.7606	p = 0.12 n≤200	NO	2	pd
413	*PLA2G4D*	p.S173G c.517A>G	No	Blood - D [1]	No fuctional domain	No/No	197/37446 (0.53%)	10/2135 (0.47%) 1 FS	0/10	Neutral (0.04)	Benign	Tolerated	0.00/55.27 (C55)	0.3458	-	NO	0.5	Neutral
413	*POLD1*	p.P146R c.437C>G	Yes	No results	Exonucelase domain	No/No	263/37786 (0.70%)	9/2137 (0.42%) 5 FS	0/9	Pathogenic (0.95)	Deleterious	Not Tolerated	0.00/102.71 (C65)	0.1793	p = 0.000042 OE	[48–51]	6.5	CGC
413	*RSBN1*	p.P148S c.442C>T	No	Blood - B [1] Liver - D [15] Colorectal - C [2] Gastric - C [22]	Pro-Rich domain	No/No	149/37401 (0.40%)	12/2137 (0.52%) 1 NS	1/12	Neutral (0.27)	Deleterious	Not Tolerated	0.00/73.35 (C65)	0.1256	p = 0.011 UE	NO	4	PD
413	*SLC13A1*	p.R277P c.830G>C	No	No results	No Pfam annotations found	No/No	241/37402 (0.64%)	15/2137 (0.70%) 1 NS	0/15	Neutral (0.02)	Benign	Tolerated	0.0/102.71 (C65)	0.6497	p = 0.064	NO	0	Neutral
413	*ZNF33A*	p.G183V c.548G>T	No	No results	No fuctional domain	No/No	166/37402 (0.44%)	4/2137 (0.19%) 1 FS	0/4	Neutral (0.12)	ND	ND		0.3098	p = 0.0081 n≤200	NO	0	Neutral
415	*LRRC66*	p.H434N c.1300C>A	No	No results	disorderSource: IUPred	No/No	276/37403 (0.74%)	7/2137 (0.33%)	2/7	Neutral (0.00)	Benign	Tolerated	0.00/68.35 (C65)	0.577	p = 0.015 n≤200	NO	0	Neutral
415	*MAMLD1*	p.A775V c.2324C>T	No	Mixed - NR [52] Colorectal - C [2]	No Pfam annotations found	No/No	172/37402 (0.46%)	17/2137 (0.79%) 2 FS/1 NS	0/17	Neutral (0.00)	ND	Tolerated	0.00/64.43 (C65)	0.7952	p = 0.23	NO	1	Neutral
415	*PIK3AP1*	p.Q285K c.853C>A	No	Blood - A [1] Colorectal - NR [2] Liver - C [15, 23]	Dof, BCAP, and BANK (DBB) motif	No/No	194/37522 (0.51%)	7/2137 (0.33%)	0/7	Pathogenic (0.98)	Deleterious	Not Tolerated	0.00/53.23 (C45)	0.1641	p = 0.17 n≤200	[53–55]	4.5	PD
415	*PIK3CA*	p.H1047L c.3140A>T	Yes	Blood - D [1] Gastric – D [22] Liver - C [23, 24] Nervous system - D [25] Skin - B [26]	PI3K/PI4K domain	Yes/Yes	10271/107457 (9.56%)	4098/15384 (26.64%)	-	Pathogenic (0.96)	Benign	Tolerated	0.00/98.69 (C65)	0	p = 0.057	Oncogene	7.5	CGC
415	*PRKD1*	p.Y800C c.2399A>G	No	No results	Protein Kinase Domain	No/No	328/38363 (0.85%)	11/2364 (0.46%) 1 NS	0/11	Pathogenic (0.98)	Deleterious	Not Tolerated	0.00/193.72 (C65)	0.0002	p = 0.075	[56–60]	2.5	pd
415	*TTC21B*	p.R898^*^ c.2692C>T	No	No results	Tetratricopeptide repeat	No/No	230/37405 (0.61%)	13/2137 (0.61%) 1 FS/1 NS	0/13	Pathogenic (0.90)	ND	ND		-	p = 0.15	NO	1.5	Neutral
416	*FAM96A*	p.E75K c.223G>A	No	Colorectal - NR [2]	Iron-sulfur cluster assembly protein	No/No	36/37402 (0.10%)	2/2137 (0.09%)	0/2	Pathogenic (0.94)	Benign	Tolerated	0.00/56.87 (C55)	0.6908	p = 0.039 N≤200	[61, 62]	1.5	Neutral
416	*FLG*	p.R1166C c.3496C>T	No	No results	disorderSource: IUPred	No/Yes	1467/37980 (3.87%)	78/2139 (3.64%) 1 NS	-	Neutral (0.01)	ND	Not Tolerated	0.00/179.53 (C65)	0.6299	p = 0.18	[63]	1.5	Neutral
416	*GRIN1*	p.Q910^*^ c.2728C>T	No	Colorectal - NR [2]	disorderSource: IUPred	No/No	162/37493 (0.43%)	7/2137 (0.33%)	0/7	Pathogenic (0.77)	ND	ND	-	-	p = 0.24	[64]	2.5	pd
416	*MYO1H*	p.E501G c.1502A>G	No	No results	Myosin motor domain	No/No	237/37317 (0.63%)	17/2126 (0.76%) 1 FS/1 NS	1/17	Pathogenic (0.98)	ND	Tolerated	0.00/97.85 (C65)	0.0825	p = 0.14 n≤200	NO	0.5	Neutral
416	*TTN*	p.L6228S c.18683T>C	No	Colorectal - B [65]	IG-Like 43 domain	No/No	4470/37491 (11.92%)	288/2105 (13.68%)	-	Pathogenic (0.81)	Deleterious	ND	-	0.1523	p = 0.019 OE	[66, 67]	4.5	PD
417	*AZI2*	p.I66V c.196A>G	No	No results	coiled_coilSource: ncoils	No/No	63/37401 (0.17%)	5/2137 (0.23%) 1 FS	0/5	Pathogenic (0.65)	Benign	Tolerated	0.00/29.61 (C25)	0.2633	p = 0.000061 n≤200	NO	0	Neutral
417	*GRHL2*	p.E32^*^ c.94G>T	No	Colorectal - D [2], pancreatic - D [58]	disorderSource: IUPred	No/No	171/37403 (0.46%)	17/2138 (0.79%) 3 NS	0/17	Pathogenic (0.99)	ND	ND	-	-	p = 0.39	[68–69]	3	pd
417	*NDST4*	p.V313F c.937G>T	No	No results	heparan sulfate-N-deacetylase domain	No/No	403/37402 (1.08%)	6/2137 (0.28%)	0/6	Pathogenic (0.98)	Deleterious	Not Tolerated	0.00/49.94 (C45)	0.0601	p = 0.1	[70, 71]	2.5	pd
417	*PRICKLE2*	p.P81L c.242C>T	No	Skin - D [26]	PET Domain	No/No	213/37402 (0.57%)	8/2138 (0.37%) 2 NS	0/8	Pathogenic (0.99)	Deleterious	Not Tolerated	0.00/97.78 (C65)	0.0751	p = 0.17 n≤200	[72, 73]	3	pd

SNVs Frequency in breast cancer^*^: frequency of SNVs (including synonymous) in breast cancer. SNVs (n) in breast cancer. Young pts/all ages: number of SNVs (excluding synonymous) in breast cancer (BC) patients ≤ 35 years/number of SNVs in BC patients with all ages (excluding patients who had unknown ages). CGC genes for which mutations have been causally implicated in cancer and which are catalogued at “Cancer Gene Census”. CCGD genes that are potential cancer drivers in genetic screens in mice and are catalogued at the “Candidate Cancer Gene Database”. NS: Nonsense; FS: Frameshift. KM-OS: (p) for overall survival evaluated through gene expression using KM plotter. Abbreviations: OE: Overexpression associated with longer survival; UE: Underexpression associated with longer survival. PD: Probably driver; pd: Possibly driver. The score system is described in Supplementary Table 9. Coments of literature is referenced in Supplementary Table 9a.

Each tumor sample was then individually explored to detect potential drivers. Three tumors presented SNVs in at least three potential cancer driver genes: 402, 406 and 415. In tumor 402, besides *PIK3CA* and *CIITA*, other candidate cancer genes harboring somatic SNVs were *CACNA1E, NES, STAB1, HECW2*, *SMURF2* and *ZNF462*. In tumor 406, SNVs were detected in three known cancer genes reported in the “Cancer Gene Census” database [19]: *PIK3CA, TP53* and *PRKAR1A*. However, the alteration detected in the latter was considered pathogenic in only one of the five mutation function assessment algorithms (Table 1). In addition, SNVs were observed in other two possibly driver genes, *IL22* and *OSR2.* In tumor 415, besides *PIK3CA*, other potential cancer drivers affected by SNVs were *PIK3AP1* and *PRKD1.*

In the other five tumors, SNVs were identified in one to three potential cancer driver genes: in tumor 413, *RSBN1*; in tumor 416: *TTN* and *GRIN1*; in tumor 401: *SEMA6D* [21, 22], as well as *MTHFD2;* in tumor 417: *GRHL2, PRICKLE2* and *NDST4*. In tumor 404, a possible cancer driver is *ELMO3*, which KM plotter indicated that overexpression is associated with poor overall survival (Supplementary Figure 1).

To further explore somatic mutations in luminal tumors (HER2 negative) from very young patients, we identified another 29 patients aged ≤35 years at diagnosis, who had data published in studies of tumor exome or genome sequencing [15–18], most of which, deposited in the COSMIC database [15–17].

In these tumors, the most frequent events were C to T transitions, representing a mean percentage of 39% of the substitutions (Supplementary Figure 2). A total of 1,617 non-synonymous variants were detected across these 29 patients, with a median number of 29 variants per patient (minimum: 9 and maximum: 546; mean: 56) (Supplementary Table 10, 10a). Some genes, that were present in our list, were also mutated in these luminal tumors, such as *PIK3CA, TP53, AK8, CIITA, FLG, POC5, POLD1*, *SEMA6D, TTN* and *LCRC66*.

Functional categories enriched in gene variants according to DAVID bioinformatics tool [23] included ATP binding, in five tumors and plasma membrane, in three tumors, among others less frequently represented (Supplementary Table 11).

**Figure 3:**
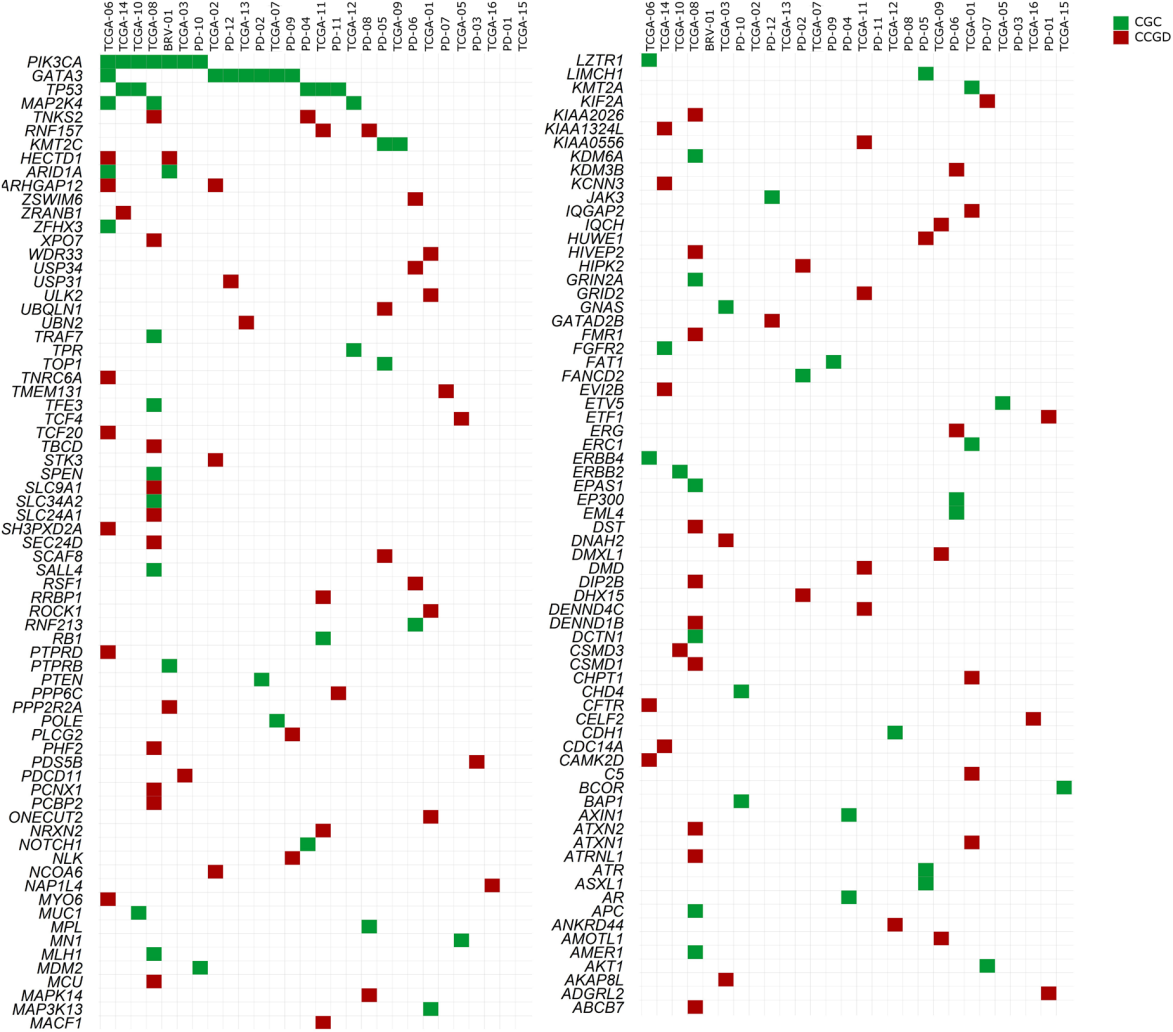
Distribution of mutated candidate driver genes among 28 tumor samples retrieved from the literature and COSMIC database All cancer genes listed at “Cancer Gene Census” (CGC) database (http://cancer.sanger.ac.uk/cosmic/census) and all driver candidates listed in “Candidate Cancer Gene Database” (CCGD), ranked as A (http://ccgd-starrlab.oit.umn.edu/about.php), are shown. Note: Sample TCGA-04 is shown exclusively in Supplementary Table 10 (but not in the figure), due to a large number of somatic mutations (CGC= 30; CCGD rank A= 56). Green: CGC; Red: CCGD, rank A [18]. Causal relationship with cancer was based on a scoring system, described in Materials and Methods. All reported genes affected by SNVs appear in Supplementary Table 10.

Seven out of these 29 tumors presented SNVs in just one cancer driver, classified in the “Cancer Gene Census” database, which were: *AKT1, MPL* (MPL Proto-Oncogene, Thrombopoietin Receptor), *TP53, GATA3* (2 samples), *BCOR* (BCL6 Corepressor) and *KMT2C* (Lysine Methyltransferase 2C), while the other 19 tumors presented SNVs in at least two cancer genes from the “Cancer Gene Census” database (Supplementary Table 12). Furthermore, three tumors did not show any variants in driver candidates from the list of “Cancer Gene Census”, but each one presented SNVs in one or two genes, already reported in the “Candidate Cancer Gene Database” category A: *PDS5B* (PDS5 cohesin associated factor); *LPHN2/ADGRL2* (Adhesion G Protein-Coupled Receptor L2) and *ETF1* (Eukaryotic translation termination factor 1); *CELF2* (CUBGBD Elav-like family member 2) and *NAP1L4* (Nucleosome assembly protein 1 like 4). All genes considered as causally implicated in cancer or potential cancer drivers are shown in Figure 3 and Supplementary Table 12. The score system (described in methods) identified *FAT2* (FAT atypical cadherin 2) as a probable driver gene in two samples, because it is a gene ranked B in CCGD, also frequently mutated in cancers and variants were considered pathogenic in three out of four prediction models of cancer causality investigated.

Among the 29 tumors, six were obtained from patients whose *BRCA1* and *BRCA2* status was known: two wild type and four mutation carriers. Somatic SNVs in both tumors from *BRCA1* and *BRCA2* wild type germline patients involved *GATA3*; however, none of the affected genes in this pair of tumors coincided with data from our patients.

**Figure 4:**
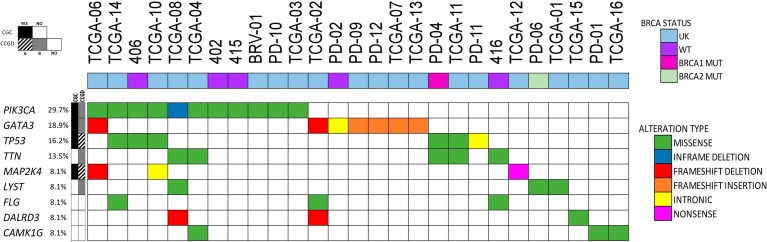
Most frequently mutated genes in luminal tumors Samples (n=27) presenting SNVs in at least one of the nine most frequently mutated genes were included (current analysis, n=4; and COSMIC Database, n=23). Type of gene alteration and *BRCA1/2* status are shown. Each column represents a single patient. UK: unknown.

Finally, we analyzed the 37 tumors all together (29 previously reported and 8 currently evaluated). Considering only SNVs detected in the genes already included in the “Cancer Gene Census” database or the “Candidate Cancer Gene Database”, categories A or B, the median number (minimum and maximum) of driver candidates per tumor, were: 2 (0-30); 2 (0-56); 2 (0-61) respectively, totalizing a median of 6 potential drivers affected per tumor (0-147) (Supplementary Tables 12). The most frequently altered cancer causing genes according to “Cancer Gene Census” were *PIK3CA* (11/37: 29.7%); *GATA3* (7/37; 18.9%), *TP53* (6/37: 16.2%) and *MAP2K4* (3/37: 8.1%). SNVs were also frequently detected in the following genes: *TTN* (7/37; 18.9%), *CAMK1G, LYST*, *DALRD3* (3/29; 10.3%) and *FLG* (3/37; 8.1%). Among these genes, it is interesting to point out that pathogenic frameshift mutations in *DALRD3* were detected in two (out of three) tumor samples. *PIK3CA* was concomitantly mutated with *TP53* in three tumors and with *GATA3* in one tumor (Figure 4; Supplementary Figure 3).

SNVs were detected in genes involved in DNA repair mechanisms in 16 out of the 37 tumors (43.2%). In 11 samples, only one gene was altered, such as *FANCD2*, *FANCL* or *BAP1*, which are involved in homologous recombination repair (HRR); *PARP4* (2 samples), involved in base excision repair (BER); *ATR* and *TP53* (the latter altered in 3 samples), involved in signaling DNA damage to cell cycle checkpoints. In two tumors, SNVs uniquely affected polymerases *POLD1* or *POLE*, which are involved in the base excision repair (BER), nucleotide excision repair (NER) and mismatch repair (MMR).

Three samples presented composite gene disturbances involving *TP53* and either *POLD1* or *RAD51* (HRR) or *POLQ* (involved in translesion synthesis, TLS). The highest number of SNVs was described in two tumors, one presenting mutations in genes involved in BER (*MUTYH)*, NER (*ERCC6*) and HRR (*PALB2*) and the other, in genes involved in MMR *(MLH1*) and HRR (*RAD9A*) [24] (Table 2).

**Table 2:** Samples with somatic mutations in genes involved in DNA repair mechanisms

Sample	Gene	Mechanisms of DNA repair							N. of variants/sample
		BER	NER	MMR	HRR	NHEJ	DDC	TLS	
**406**	*TP53*						X		7
**413**	*POLD1*	X	X	X					6
**PD-02**	*FANCD2*				X				17
**PD-04**	*POLD1*	X	X	X					55
	*TP53*						X		
**PD-05**	*ATR*					X	X		36
**PD-06**	*FANCL*				X				76
**PD-10**	*BAP1*				X				17
**PD-11**	*TP53*						X		16
**TCGA-01**	*PARP4*	X							46
**TCGA-04**	*MUTYH*	X							546
	*ERCC6*		X						
	*PALB2*				X				
**TCGA-06**	*PARP4*	X							48
**TCGA-07**	*POLE*	X	X	X					21
**TCGA-08**	*MLH1*			X					229
	*RAD9A*				X				
**TCGA-10**	*TP53*						X		9
**TCGA-11**	*TP53*						X		84
	*RAD51*				X				
**TCGA-14**	*POLQ*							X	79
	*TP53*						X		

DNA repair genes altered and respective pathways affected per patient. Base excision repair (BER), nucleotide excision repair (NER), mismatch repair (MMR), homologous recombination repair (HRR), non-homologous end-joining (NHEJ), DNA damage signaling to cell cycle checkpoints (DDC) and translesion synthesis (TLS).

In addition, variants involving 213 genes were of nonsense or frameshift types (Supplementary Table 12). One of these genes, *RBM16/SCAF8* is a driver candidate, because it is also listed in the “Candidate Cancer Gene Database”, rank A, in at least two solid tumor types. Among these genes, 42 were involved in positive regulation of gene expression [25] and one of these genes was mutated in 15 different samples and more than one gene was mutated in five other samples. Hence, 54% of the luminal samples presented at least one mutated gene involved in gene expression regulation (Table 3).

**Table 3:** Characterization of nonsense and frameshift variants according to CCGD A/B and biological function (Toppgene) per sample

Sample	Gene ID	CCGD A	CCGD B	Positive regulation of gene expression	N. of variants/sample
**415**	*TTC21B*	-	-	-	6
**416**	*GRIN1*	-	-	*GRIN1*	5
**417**	*GRHL2*	-	-	*GRHL2*	4
**BRV-01**	*CTCFL*; *FAM118B*; *MARVELD2*; *VPS11*	-	-	*CTCFL*	28
**PD-02**	*PTEN*	PTEN	-	*PTEN*	17
**PD-04**	*FSCB*; *IL12RB2*; *AKAP11*	-	-	-	55
**PD-05**	*ATR*; *VIM HK1*; *OR7C1*; *RBM16*/*SCAF8*	*ATR; RBM16/SCAF8*	-	*VIM*	36
**PD-06**	*CACNA2D3*; *DNAH17*; *PEX5L MAB21L3*; *SYNE1*; *ZMYND11*	-	*ZMYND11*; *DNAH17*	-	76
**PD-07**	*PTRH1*	-	-	-	13
**PD-08**	*KCNJ15*; *SYTL2 ENSG00000233280*	-	-	-	41
**PD-09**	*PLCG2 SHCBP1*; *GATA3*	*PLCG2*	*GATA3*	*GATA3*	15
**PD-10**	*NEMF*		*NEMF*	-	17
**PD-11**	*KRTAP2-1*; *SLC2A3*; *NARG2*/*ICE2 COL22A1*	*-*	*-*	*NARG2/ICE2*	16
**PD-12**	*GATA3*; *PDE7A*	*-*	*GATA3*	*GATA3*	22
**TCGA-01**	*ABCA10*; *NTRK2*; *MAP3K6_ENST00000374040*; *CX3CR1*; *KBTBD4*; *KMT2A*	*-*	*-*	*KMT2A; NTRK2*	46
**TCGA-02**	*GATA3*; *DALRD3*; *RASGRP2*; *SALL3*; *TNFSF9*	*-*	*GATA3*	*GATA3*	18
**TCGA-03**	*C1orf187*; *NR1I3*; *FAM155A*; *GNAS; PCDHA2*; *SSC5D*; *SEC14L5*; *WDR81_ENST00000409644*	*-*	*-*	*NR1I3*	21
**TCGA-04**	41	*UBR5*	*BTBD7; ITGB1; KLHDC2; MTA2; ODF2; PCCA; PPFIA3; RASGRF1*	*ITGA8; MTA2; NFKBIA; ATF7IP; SPAG8; TARBP2; TLR3*	546
**TCGA-05**	*NMS*; *FAM111B; DYNC2H1_ENST00000398093*	*-*	*-*	*-*	31
**TCGA-06**	*ARID1A*; *CFTR*; *SYNM*; *GATA3*; *CCDC61*; *CDK18*; *IRF7*; *TCF20*; *KIAA0430/MARF1*; *LZTR1*; *MAP2K4*; *SH3PXD2A*	*ARID1A; TCF20; CFTR; MAP2K4; SH3PXD2A;*	*GATA3*	*ARID1A; GATA3; IRF7; TCF20*	48
**TCGA-07**	*GATA3*		*GATA3*	*GATA3*	21
**TCGA-08**	73	ATXN2; DIP2B; KIAA2026_E NST0000039 9933; PCNX1; PHF2; TNKS2; SLC9A1	CLMN; KIAA0947_ ENST0000 0296564/ICE1; OSBPL1A; RAB11A; ARHGAP2 9; SLTM;	17	229
**TCGA-09**	*DYNC1H1*; *IGSF3*; *MAST1*; *AKAP12*; *ASB10_ENST00000422024*; *NASP*; *TENM1/ODZ1*; *THOC5*; *ZNF799*	-	*THOC5*	*THOC5*	35
**TCGA-10**	*C9orf66*	-	*-*	*-*	9
**TCGA-11**	*A2M*; *CHKB*; *NBR1*; *RB1*; *SYT3*; *ARR3*; *KIFC3*; *PPP1R3C*; *ZBTB24*	*RB1*	*NBR1*	*RB1*	84
**TCGA-12**	*EFEMP1*; *MAP2K4*; *C1orf35*; *KIF26A*	*MAP2K4*	*-*	*-*	15
**TCGA-13**	*SNUPN_ENST00000371091*; *GATA3*; *GRM6*	-	*GATA3*	*GATA3*	13
**TCGA-14**	*PRDM5*; *COL14A1*; *POLA1*; *SLC22A25*	-	*PRDM5*	*-*	79
**TCGA-15**	*IGSF1*; *NFYB*; *SCN2A*; *TRAF5*	-	*NFYB*	*NFYB*; *TRAF5*	34
**TCGA-16**	*JHDM1D*/*KDM7A*	-	*-*	*JHDM1D/KDM7A*	30

Samples with genes affected by nonsense or frameshift variants were searched for candidate cancer genes (CCGD database ranks A or B) and involvement in positive regulation of gene expression (GO: biological process).

## DISCUSSION

Our goal was to characterize *BRCA1* and *BRCA2* germline mutations in a group of very young Brazilian patients and to identify somatic mutations in luminal HER2 negative breast cancer.

Our data indicates that in very young Brazilian patients, *BRCA1* and *BRCA2* mutation frequency is 16%, similar to that already reported in comparable groups of patients from Brazil [7], as well as from other countries [4–8]. However, there is still a lack of information regarding the spectrum of mutations and VUS in the average Brazilian population, that harbors peculiar characteristics of miscegenation, comprehending a mixture of 70% European, 15% African and 15% Amerindian ancestry genes [26]. In our patients we could detect a new mutation in the *BRCA2* gene, as well as another 13 variants of unknown significance.

Somatic mutation in the group of eight luminal samples (HER2 negative) from *BRCA1* and *BRCA2* wild type carriers were then investigated. The overall mutation rate in these tumor samples was 1.93 per Mbp, as compared with 1.18 per Mbp and 1.66 per Mbp reported in luminal samples from post-menopausal women [27] and other breast cancer samples in general, irrespective of subtype or age [18], respectively. We have also detected a predominance of C>T substitutions, a signature previously associated with advancing age, indicating that these alterations are also the most prevalent in early onset breast cancer [28]. In accordance, the same signature was also the most frequent among other luminal tumors from very young patients deposited in COSMIC [15–17].

In the present series, somatic SNVs affected, among others, five known cancer causing genes, *PIK3CA, TP53, PRKAR1A, POLD1* and *CIITA* [19]. *PIK3CA* was the only recurrent finding, which was detected in three different tumors. Other cancer causing candidates were *SMURF2*, *PIK3AP1, RSBN1, TTN* and *SEMA6D*, which were ranked in the top 25% potential drivers in transposon insertional mutagenesis studies in mice [21, 29]. These genes variants were also considered pathogenic/deleterious/not tolerated in at least two out of five mutation function assessment algorithms. In addition, SNVs were detected in genes that were previously associated with cancer, such as *CACNA1E, PRKD1, NDST4*, and were also considered pathogenic/deleterious/not tolerated in at least three mutation function models. Moreover, nonsense mutations were detected in *GRHL2*, *GRIN1*, *NOL9* and *TTC21B*, however only *GRHL2* and *GRIN1* were previously shown to be involved in cancer.

*GRHL2* (grainyhead-like transcription factor 2), is a transcription factor that mainly suppresses epithelial mesenchymal transition (EMT) process. It is considered a potential tumor suppressor gene in breast cancer [30]. *GRIN1* or *NMDAR1* (N-Methyl-D-Aspartate Receptor Subunit NR1) was shown to be expressed in breast cancer specimens, but not in normal breast and to be involved in tumor growth [31], being thus, a potential oncogene. *SMURF2* (SMAD specific E3 ubiquitin protein ligase 2) is a tumor suppressor involved in the maintenance of genomic stability and suppression of breast cancer cells invasiveness [32, 33]. *PIK3AP1* (phosphoinositide-3-kinase adaptor protein 1), also known as *BCAP*, is involved in the phosphatidylinositol 3-kinase (PI3K) pathway and genome wide association studies suggest that the *PIK3AP1* gene region might be involved in breast cancer predisposition [34]. *RSBN1* encodes a round spermatid basic protein 1, which function is not well established. In breast cancer lineages *RSBN1* expression is induced by hypoxia and the gene is a potential HIF target [35]. Besides, in luminal breast cancer, *RSBN1* high expression is associated with a better prognosis in luminal breast cancer [20].

*CACNA1E*, calcium voltage-gated channel subunit alpha-1 E, was shown to be underexpressed in breast cancer compared with normal tissue and was hypothesized to be a tumor suppressor gene in some types of cancer [36]. In the current study, *CACNA1E* mutation occurred in a hot spot site already reported as altered in at least five different types of cancers. *PRKD1* codes for a serine-threonine kinase and mutations all over the gene were described in various types of cancer. A recurrent activating mutation in the kinase domain described in polymorphous low grade adenocarcinoma of salivary glands, was associated with improved metastasis free survival in a transfection cell model [37]. In breast cancer cells however, *PRKD1* may display a dual function as an oncogene, stimulating drug resistance in breast cancer stemness [38] or as a tumor suppressor, blocking invasion and metastasis. In our patient, *PRKD1* mutation was located in the distal region in the kinase domain.

*POLD1* codes for the catalytic subunit of DNA polymerase delta, which plays a role in DNA replication and DNA repair [39]. Both germline and somatic gene mutations may cause an ultra-mutated phenotype, and mutations affecting the exonuclease domain are associated with high risk of colorectal and endometrial carcinomas [40]. In our patient, *POLD1* amino-acid change occurred in the exonuclease domain. In addition, *POLD1* was also mutated in another luminal sample from a very young patient present in COSMIC database [16]. Although infrequent in breast cancer, five of ten *POLD1* somatic mutations reported in the COSMIC database were of frameshift nature, therefore, potentially pathogenic (http://cancer.sanger.ac.uk/cosmic) (July, 2017).

*NDST4* (N-deacetylase/N-sulfotransferase-4), is involved in heparan sulfate (HS) biosynthesis and may be implicated in positive or negative aspects of tumor progression. In colorectal cancer, *NDST4* loss of function was implicated in tumor progression and the gene was considered a candidate tumor suppressor [41].

*ELMO3* (Engulfment and Motility 3) is involved in induction of cell proliferation, invasion and metastasis in colorectal cancer cells [42]. In addition, *ELMO3* positive/higher expression is associated with poor overall survival in non-small cell lung cancer and head and neck cancer, as well as in breast cancer, corroborating its role as an oncogene [43, 44]. *MTHFD2* (methylenetetrahydrofolate dehydrogenase (NADP+ dependent) 2) is a source of carbon units for purine synthesis in rapidly growing cancer cells and has been associated with poor prognosis in patients with breast cancer [45, 46]. *SEMA6D* (Semaphorin 6D) encodes a transmembrane protein and its overexpression increases proliferation and tumor formation, playing an oncogene role in osteosarcoma [47]. *SEMA6D* high expression is also associated with better patient survival, especially among triple negative breast cancer [48].

The results, considering all the 37 tumors (29 previously analyzed and the eight currently analyzed), suggest that the median number of driver candidates per tumor is six, however, this number is quite variable. Moreover, in luminal tumors from very young patients the most frequent cancer drivers are *PIK3CA*; *GATA3*, and *TP53.* In accordance with a recent analysis that included some of these very young patients (≤ 35 years) but mainly older patients, with ages up to 45 years, the most prevalent mutated genes were also *PIK3CA, TP53, GATA3* and *TTN* [49].

Other genes frequently mutated were *CAMK1G* (Calcium/Calmodulin Dependent Protein Kinase IG), *DALRD3* (DALR Anticodon Binding Domain Containing 3), *LYST* (Lysosomal Trafficking Regulator) and *MAP2K4* (3/37: 8.1%). *DALRD3* contains two microRNA (miRNA) precursors (miR-191 and miR-425) in one of its introns and the expression of both microRNAs is higher in estrogen receptor alpha (ER) positive cells. However, estrogen regulation of miR191/425-*DALRD3* transcriptional unit is complex and may be unparalleled. Although the exact function *DALRD3* is not known, in estrogen receptor positive cells, miR-191/425 works as oncogenes by inducing proliferation. Interestingly, SNVs in *DALRD3* detected in two out of three samples from young patients were of the frameshift kind [50]. *LYST* gene silencing may inhibit cell proliferation and induce apoptosis in myeloma cells [51].

It is worth mentioning that somatic mutations in genes involved in DNA repair mechanisms were quite common and any of these pathways might be altered: base excision repair (BER), nucleotide excision repair (NER), mismatch repair (MMR), homologous recombination repair (HRR) as well as signaling DNA damage to cell cycle checkpoints. The highest number of SNVs was described in two tumors presenting mutations in genes involved in HRR, as well as in other DNA repair mechanisms concomitantly [24]. In accordance, an association between younger age at diagnosis and risk genotypes for genes involved in DNA repair, such as NER, MMR and NHEJ (Non-homologous end-joining) have been already reported [52].

The weaknesses and the strengths of our study involve the number of exomes analyzed, though small, add around 20% of samples to the available data thus far.

In summary, in luminal tumors (HER2 negative) from very young patients, the most frequent events were C to T transitions. SNVs were detected in a median number of six potential driver genes per sample, and 43% of the tumors presented mutations in DNA repair genes and 54% of the tumors presented at least one pathogenic mutation in a gene involved in positive regulation of gene transcription. The most frequent somatic mutations involved cancer driver genes, such as *PIK3CA, TP53* and *GATA3*. Other potential driver candidates currently identified were *GRHL2, PIK3AP1, CACNA1E* and *SEMA6D*.

## MATERIALS AND METHODS

### Patients

This study was approved by the Institutional Ethics Committee of Instituto Brasileiro de Controle do Câncer (IBCC) and Instituto do Câncer do Estado de São Paulo (ICESP)/Faculdade de Medicina da Universidade de São Paulo (FMUSP). All patients were informed and signed an informed consent.

Early onset breast cancer was defined as a disease diagnosed in very young women aged ≤35 years. No patients received previous medical treatment for their breast cancer before the tumor collection through biopsy or mastectomy procedures.

Patients were interviewed for family history suggestive of Hereditary Breast and Ovarian Cancer Syndrome (HBOCS) in close relatives, such as first, second, and third degree family members. Family history was considered informative if the patient could report on at least two first or second degree female relatives having lived beyond age 45 in both parental lineages, otherwise it was considered unknown or limited (National Comprehensive Cancer Network, NCCN, https://www.nccn.org/professionals/physician_gls/pdf/genetics_screening.pdf, (February 2012). Genetic/Familial high-risk assessment: breast and ovarian. Patients were also asked about their ancestry, to obtain information of country or continent where their parents and grandparents (at least) were born.

The median age of the 79 patients at diagnosis was 32 years, most of whom diagnosed with invasive ductal carcinoma (91.1%), high histological grade (48%), Ki67 >14% (90.4%), luminal subtype (65.8%; ER and/or PR positive and HER2 negative), and advanced stage disease (clinical stages III/IV; 47.1%) (Supplementary Table 1). HER2 positivity was defined as immunohistochemistry 3+ or 2+, the latter, associated with Fluorescence *in situ* hybridization (FISH)-amplification. HER2 immunohistochemistry and FISH were scored according to ASCO/CAP guidelines [53].

All women had a blood sample collected for *BRCA1* and *BRCA2* whole gene sequencing (see below).

Among the 79 women, 12 had fresh-frozen tumor samples collected during breast surgery. Among the latter, eight patients, who were *BRCA1* and *BRCA2* wild type carriers bearing luminal HER2 negative tumors, had their samples subsequently analyzed through whole exome sequencing (see below) (Supplementary Figure 4).

### DNA extraction from blood and tumor tissue

DNA was extracted from 8mL of whole blood using the Kit Illustra Blood GenomicPrep Mini Spin Kit (GE Healthcare Bio-Sciences, Pittsburgh, PA, USA/28-9042-64); and from cancer cells enriched areas from fresh-frozen or FFPE samples, using the QIAamp DNA Mini Kit - Qiagen (Qiagen, Valencia, CA, USA/51304) and QIAamp^®^ DNA FFPE Tissue (Qiagen/56404), respectively, following instructions of the manufacturer.

### Direct sequencing of *BRCA1* and *BRCA2* genes

#### Polymerase chain reaction (PCR) amplification and sanger sequencing

Briefly, the complete coding region of *BRCA1* (U14680 or NM_7294.2) and *BRCA2* (U43746 or NM_000059.1) genes were amplified and sequenced in both forward and reverse directions. Primers and conditions are described in Supplementary Table 13 for *BRCA1* [54, 55] and Supplementary Table 14 for *BRCA2* [56]. Sequences obtained were visualized by Chromas (v2.33; Technelysium Pty, Ltd Eden Prairie, MN, USA) and by Mutation Surveyor software (v3.20, SoftGenetics LLC, State College, PA, USA). If a pathogenic mutation was identified, a new DNA sample derived from a second venipuncture was resequenced for confirmation. Full details of methods are given in the Supplementary Methods.

#### Multiplex ligation-dependent probe amplification (MLPA) of *BRCA1* and *BRCA2* genes

Samples from patients, who were negative for *BRCA1* and *BRCA2* pathogenic mutations were investigated for large deletions and duplications, using the MLPA commercial kits *SALSA*^®^ *MLPA*^®^ *P002 BRCA1 probemix* (P002 - 100R) and *SALSA*^®^ *MLPA*^®^ *P045 BRCA2/CHEK2 probemix* (P045 - 100R) (MRC-Holland, Amsterdam, The Netherlands), as described in Supplementary Methods. Sequencing to detect the presence of *CHEK2* hot spot (c.1100delC) was also performed.

### *BRCA1* and *BRCA2* sequencing analysis and reporting criteria

All sequence variants were named according to nomenclature used by The Human Gene Mutation Database, HGMD (http://www.hgmd.cf.ac.uk/ac/index.php). The variants were searched for their classification in five publicly accessible databases: Breast Cancer Information Core (BIC) [57], Leiden Open Variation Database (LOVD v3.0 build 13), [58], Leiden Open Variation Database - International Agency for Research on Cancer (LOVD-IARC v.2.0 Build 22), Universal Mutation Database (UMD), [59, 60], and ClinVar [61], this search was performed on the months of April - June 2017.

Gene variants were submitted to the following *in silico* prediction models: Polymorphism Phenotyping (PolyPhen; v2.2.2) [62], Sorting Intolerant From Tolerant (SIFT; v1.0.3) [63], Align-GVGD [64, 65], for missense variants; Protein Variation Effect Analyzer (Provean; v1.1) [66] for in-frame deletions, and Human Splicing Finder [67] to check for intronic and exonic variants leading to potential splicing defects.

Minor allele frequency was checked in the 1000 Genomes Project database [68], the Exome Aggregation Consortium (ExAC) [69, 70], the Global MAF dbSNP [71], and the Exome Variant Server, NHLBI GO Exome Sequencing Project (ESP) [72].

The variants were classified according to recommendations of the American College of Medical Genetics and Genomics in: pathogenic, likely pathogenic, benign, likely benign and variant of uncertain significance (VUS) [73]. Variants for *BRCA1* were also checked for co-occurrence with known pathogenic mutations in the same patient. If VUS were classified in two of the five databases, and categorized as benign (BIC and ClinVar), no known pathogenicity (LOVD), 1-not pathogenic (LOVD-IARC), or 1-neutral (UMD), they were reclassified as benign.

### Exome sequencing

DNA extracted from mononuclear cells and fresh tumor samples (containing at least 70% malignant cells) from eight patients was used to prepare a DNA library with the Illumina Nextera Rapid Capture Expanded kit (Illumina, Inc., San Diego, CA, USA/FC-140-1004), as detailed in Supplementary Methods. Shortly, genomic DNA (gDNA) was enzymatically fragmented while tags were simultaneously added. After purification, a limited-cycle PCR program was performed to ligate adapters and amplify libraries. Once gDNA libraries were prepared, exon-specific capture probes attached to streptavidin beads were used to enrich fragments containing only regions of interest, comprising 201,121 exons, totaling 62 mega base pairs (Mbp) of the genome. Exome libraries were then evaluated on a DNA 1000 Agilent 2100 Bioanalyzer chip (Agilent Technologies, Santa Clara, CA, USA) and quantified using KAPA SYBR FAST qPCR Kits (Kapa Biosystems, Wilmington, MA, USA, part #KK4602) prior to cluster generation. Pooled libraries were loaded on six lanes of one flow cell and sequenced on HiSeq 1000 platform (Illumina, Inc.) using 2 × 100bp paired-end reads, with a median of 95.3% of targeted bases covered at least 30-fold across the sample set.

### Exome sequencing analysis

BWA (v0.5.7) [74] software was used to align 8 paired tumor/blood exome samples, using hg19 as the reference genome and Picard (v1.92) to mark duplicates. Paired tumor-normal samples were processed together using GATK (v2.4.9) [75] for local realignment and for base quality recalibration. SAMtools (v0.1.9) and Picard (v1.107) were then used to process the bam headers and to index the samples, respectively [76].

To detect somatic single nucleotide variants (SNVs), SomaticSniper (v1.0.2) [77] was utilized. Default parameters were used to call SNVs, except for the mapping quality threshold, which was set to 1, as recommended by the developer. Standard, LOH, bam-readcount, false positive and lastly high confidence filters were applied using SAMtools (v0.1.6) and scripts provided by the SomaticSniper package. The final VCF file, containing high-confidence somatic SNVs, was used in downstream analyses.

An in-house perl- and R- based pipeline was used to identify recurrent mutations. Parameters were set to find genes that were mutated in at least 2 samples. This pipeline uses lists of SNPs compiled from various studies to filter out likely false positive SNPs from the samples, unless they are found in the Catalogue of Somatic Mutations in Cancer (COSMIC v71) database for coding and non-coding mutations [78]. After somatic SNVs were called using SomaticSniper, the SNPs were annotated by ANNOVAR (v2014-07-14) [79], using the RefGene database. Nonsynonymous, stop-loss, stop-gain and splice-site SNVs (based on RefGene annotations) were considered functional. SNVs were filtered using tabixpp (3b299cc) [80], removing SNVs found in any of the following databases: Fuentes, 2012 [81], dbSNP142 [82], 1000 Genomes Project (v3) [68], AccuSNP blacklist (invalidated SNVs from 68 human colorectal cancer exomes (in preparation), generated from GATK (v2.4.9 UG) and AccuSNP platform (Roche NimbleGen) analyses), and ENCODE DAC and Duke [83]. SeqSig (v3.6.4)[84] was used to identify likely driver non-synonymous mutations. This test assumes that for each patient, mutations are independent among nucleotides and homogeneous across all positions on coding regions and compute the background mutation rate for non-synonymous mutations. It uses the convolution law and may be used in situations where samples are not abundant. Discrepancies between the number of genes found in Supplementary Table 15 and that plotted in Figure 2, are due to the collapsing of variants into genes. SnpEff (v4) [85] was then used to predict amino acid changes. Data visualization used the BPG package (v5.2.1) in R [86].

### Analysis of somatic variants to identification of candidate driver genes

Genes candidates were then searched for in the “Cancer Gene Census” (CGC) database (http://cancer.sanger.ac.uk/census/) [19] to identify genes causally implicated in cancer, as well as in the “Candidate Cancer Genes Database” (CCGD) (http://ccgd-starrlab.oit.umn.edu/search.php) [21], to identify potential cancer drivers, detected in mouse insertional mutagenesis experiments. In this model, candidate genes were associated with common insertion sites (CIS), which were ranked based either on the number of insertions or the p-value: A for the top 10%; B for the top 11-25%, C for the top 26-50% and D for the bottom 50%. CISs identified in screens that did not include insertion numbers or p-values are denoted as Not Ranked [21]. Afterwards, gene mutations were analyzed through mutation function assessment algorithms: PolyPhen, SIFT, Align GV/GD [62–65], Functional analysis through Hidden Markov Models (FATHMM; v2.3) (http://fathmm.biocompute.org.uk/) [87], and Cancer-Related Analysis of Variants Toolkit (CRAVAT), [88]. This search was performed between April and June 2017, and the latter three algorithms were reviewed in December 2017.

We have then developed a scoring system in order to identify potential cancer drivers. The genes found in CGC were scored 3 points; CCGD was scored according to the highest rank for each sample: “A”: 2 points, “B”: 1.5 points, “C”: 1.0 point, “D”: 0.5 point; “Not Ranked” variants were not scored; mutation domain, frequency of the variant in other cancers and/or in breast cancer (≥1%) were scored 0.5 point each; mutation consequence when nonsense or frameshift was scored 1.5 points; mutation function assessment algorithms FATHMM, PolyPhen, SIFT, GV/GD and CRAVAT-CHASM (3.0) were scored by 1 point, if the variant was considered pathogenic at least in 3 of them.

### Analysis of somatic variants identified in other published manuscripts and COSMIC database

For this analysis, publicly available data about 29 patients, aged 35 years or younger, was obtained.

Most patients (n=28) had data for tumor exome or genome sequencing deposited in the COSMIC database [15–17] (TCGA, 2012, n=16; Nik-Zainal *et al.*, 2016, n=9; Stephens *et al.*, 2012, n=3). Additionally, data for one patient was recovered from a published manuscript [18], which was not available in COSMIC. Only HER2 negative tumors were included. One and four of these patients were *BRCA1* and *BRCA2* mutation carriers, respectively. *BRCA* mutation status of the remaining patients was unknown [16, 17].

For the present analysis, among the total number of mutations per patient, repeated substitutions detected in the same chromosomal position were considered only once. In addition, only non-synonymous mutations were contemplated.

The list of nonsynonymous variants derived from each tumor was then clustered using the DAVID v6.7 bioinformatics tool (The Database for Annotation, Visualization, and Integrated Discovery) [23], in order to explore its biological meaning. Only one Gene Ontology category (p ≤0.05) or Interpro process (in the absence of GO category) was selected for each tumor sample. If more than one GO category was enriched, the one containing the largest number of genes was chosen.

To identify potential cancer driver genes a scoring system has been developed. The genes found in CGC were scored 3.0 points; CCGD was scored according to the highest rank for each sample: “A”: 2 points, “B”: 1.5 points, “C”: 1.0 point, “D”: 0.5 point; “Not Ranked” variants were not scored; mutation domain, frequency of the variant in other cancers and/or in breast cancer (≥1%) were scored 0.5 point each; mutation consequence when nonsense or frameshift was scored 1.5 points; mutation function assessment algorithms FATHMM, PolyPhen, SIFT and CRAVAT-CHASM (3.0) were scored by 0.5 point, if the variant was considered pathogenic at least in 2 of them; were scored by 1 point, if the variant was considered pathogenic in 3 or 4 of them. Gene variants scoring ≥3.5 were considered as candidates for cancer drivers (Supplementary Table 10, 10a).

The search in the referred databases and prediction tools was performed for this analysis until December, 2017.

Toppgene was used to identify biological processes enriched in the list of genes affected by pathogenic mutations (nonsense and frame shift). (https://toppgene.cchmc.org/enrichment.jsp). Gene ID followed by ENST number was searched using the gene ID without ENST number.

Ten functions (biological process) presented more than 10 affected genes and had a p value, Bonferroni and FDR <0.05, including positive regulation of gene expression. Analysis was performed in March 2018.

## SUPPLEMENTARY MATERIALS FIGURES AND TABLES




