# Supplementary material for: Somatic mutations in early onset luminal breast cancer
Source: Oncotarget. 2018 Apr 27;9(32):22460–79. doi: 10.18632/oncotarget.25123 (PMC5976478; doi:10.18632/oncotarget.25123)
Supplement: Supplementary file 1 [file oncotarget-09-22460-s001.pdf]

Somatic mutations in early onset luminal breast cancer

SUPPLEMENTARY MATERIALS

Direct sequencing of *BRCA1* and *BRCA2* genes

Polymerase chain reaction (PCR) amplification and sanger sequencing

The complete coding region of *BRCA*1 (U14680 or NM_7294.2) and *BRCA2* (U43746 or NM_000059.1), including 50-100 base pairs (bp) of non-coding sequences, flanking the 5' and 3' ends of each exon, were amplified by PCR using 33 pairs of primers for *BRCA1* gene and 48 pairs of primers for *BRCA2* gene 0.4 μM of each primer. Furthermore, the PCR reactions contained AmpliTaq Gold enzyme 250U (final concentration 0.4U/μl) (Applied Biosystems, Foster City, CA, USA); *AmpliTaq Gold* buffer 1X; Magnesium Chloride (1.5 mM to 3.0 mM); deoxynucleotides (dNTPs) (final concentration 0.16 mM) (Invitrogen, Carlsbad, CA, USA - AM8200) and 50 ng DNA, for a final reaction volume of 10 μl, in a GeneAmp 9700 Thermal Cycler (Applied Biosystems). Fragments of *BRCA2* exon 11 were amplified by touchdown PCR. Primers and conditions are described in Supplementary Table 2 (*BRCA1*) and Supplementary Table 3 (*BRCA2*).

Each PCR product was treated with *Illustra™ ExoStar™ 1-Step* (GE Healthcare Bio-Sciences, Pittsburgh, PA, USA) and incubated at 37°C for 15 min, followed by 80°C for 15 min, to remove unincorporated primers and dNTPs. Afterwards, all PCR products were sequenced in both forward and reverse directions using BigDye® Terminator v3.1 (Applied Biosystems, Foster City, CA, USA - 4337456), according to the manufacturer's instructions and after treated using *BigDye XTerminator® Purification Kit* (Applied Biosystems - 4376487). The final product was sequenced on a 3500 Genetic Analyzer (Applied Biosystems - Hitachi). Sequences obtained were visualized by Chromas (v2.33; Technelysium Pty, Ltd Eden Prairie, MN, USA) and by Mutation Surveyor (v3.20, SoftGenetics LLC, State College, PA, USA).

Multiplex ligation-dependent probe amplification (MLPA) of *BRCA1* and *BRCA2* genes

At first, 50 ng of genomic DNA in 2,5 μl ultrapure water was denatured for ten minutes at 98°C after which 1,5 μl of the probemix mixture was added (0,75 μl of MLPA probe and 0,75 μl of MLPA buffer). The sample DNA and probemix mixture were heated at 95°C for 1 minute and incubated at 60°C overnight (17 h). Afterwards, ligation was performed with 1,5 μl of ligase buffer A, 1,5 μl of ligase buffer B, 0,5 μl of Ligase-65 and 12,5 μl of water and maintained at 54°C for 15 minutes. Then ligase was inactivated by incubation for five minutes at 98°C. Amplification was performed by adding 5 μl Mix Polymerase (1 μl of SALSA PCR primers, 0,25 μl of SALSA polymerase and 3,75 μl of water) and heated at 95°C for 1 minute. PCR was carried out for 35 cycles (30 secs at 95°C, 30 secs at 60°C and 60 secs at 72°C) followed by 20 minutes at 72°C in a GeneAmp 9700 Thermal Cycler (Applied Biosystems). Afterwards, 1 μl of PCR product was diluted 1:10 in water, 0,075 μl *GeneScan™ 600 LIZ® dye Size Standard v2.0* (*Applied Biosystems* - 4408399) and 9 μl Hi-Di Formamide (Applied Biosystems - 4440753) and incubated at 80°C for 2 minutes in a GeneAmp 9700 Thermal Cycler (Applied Biosystems). The fragments were analyzed on an *Applied Biosystems 3500 Genetic Analyzer* (*Applied Biosystems - Hitachi*) and analysis was performed using Coffalyser. NET *software* (MRC Holland, Amsterdam, Netherlands).

Exome sequencing

50 ng gDNA from tumor and blood were used to prepare exome libraries using the Illumina Nextera Rapid Capture Expanded kit (Illumina, Inc., San Diego, CA – USA/FC-140-1004) according to the following steps:

1. Tagmentation of Genomic DNA: the genomic DNA was tagmented (tags and fragments) using Nextera transposome. The Nextera transposome simultaneously fragments the genomic DNA and adds adapter sequences to the ends, allowing amplification by PCR in subsequent processes. Next the tagmented DNA was purified.

2. First PCR Amplification: the purified tagmented DNA was amplified via a limited-cycle PCR program. Barcodes index 1 (i7) and index 2 (i5) needed for sequencing, as well as common adapters (P5 and P7) required for cluster generation and sequencing were then added. After wards, beads were used to purify the library DNA and remove unwanted products.

3. First Hybridization: the DNA library was mixed with capture probes to targeted regions of interest. Furthermore, the samples were organized in two pools of eight samples each, using different indices.

4. First Capture: the probes hybridized to the targeted regions of interest were captured using streptavidin beads. Two heated wash procedures were used to remove nonspecific binding from the beads. The enriched libraries were then eluted from the beads and prepared for a second round of hybridization.

5. Second Hybridization: the eluted DNA libraries from the first enrichment round were combined with additional capture probes to targeted regions of interest. This second hybridization was required to ensure high specificity of the captured regions.

6. Second Capture: the probes hybridized to the targeted regions of interest were captured using streptavidin beads. Two heated wash procedures were used to remove nonspecific binding from the beads. The enriched libraries were then eluted from the beads and prepared for a second round of hybridization. At the end of this process, captured library DNA was purified using beads.

7. Second PCR Amplification: the captured libraries were amplified via a limited-cycle PCR program. Afterwards, beads were used to purify the enriched library DNA and remove unwanted products.

The exome capture libraries were quality-controlled using an Agilent 2100 Bioanalyzer (Agilent Technologies, Santa Clara, CA, USA), and quantified using the KAPA SYBR FAST qPCR Kits (Kapa Biosystems, Wilmington, MA, USA, part #KK4602) prior to cluster generation on an Illumina cBot. Pooled libraries were loaded on six lanes of one flow cell, using 2x100bp paired-end reads and sequenced on an Illumina HiSeq 1000 platform, targeting 201,121 exons, comprising 62 mega base pairs (Mbp) of the genome and with a median of 95.3% of targeted bases covered at least 30-fold across the sample set.

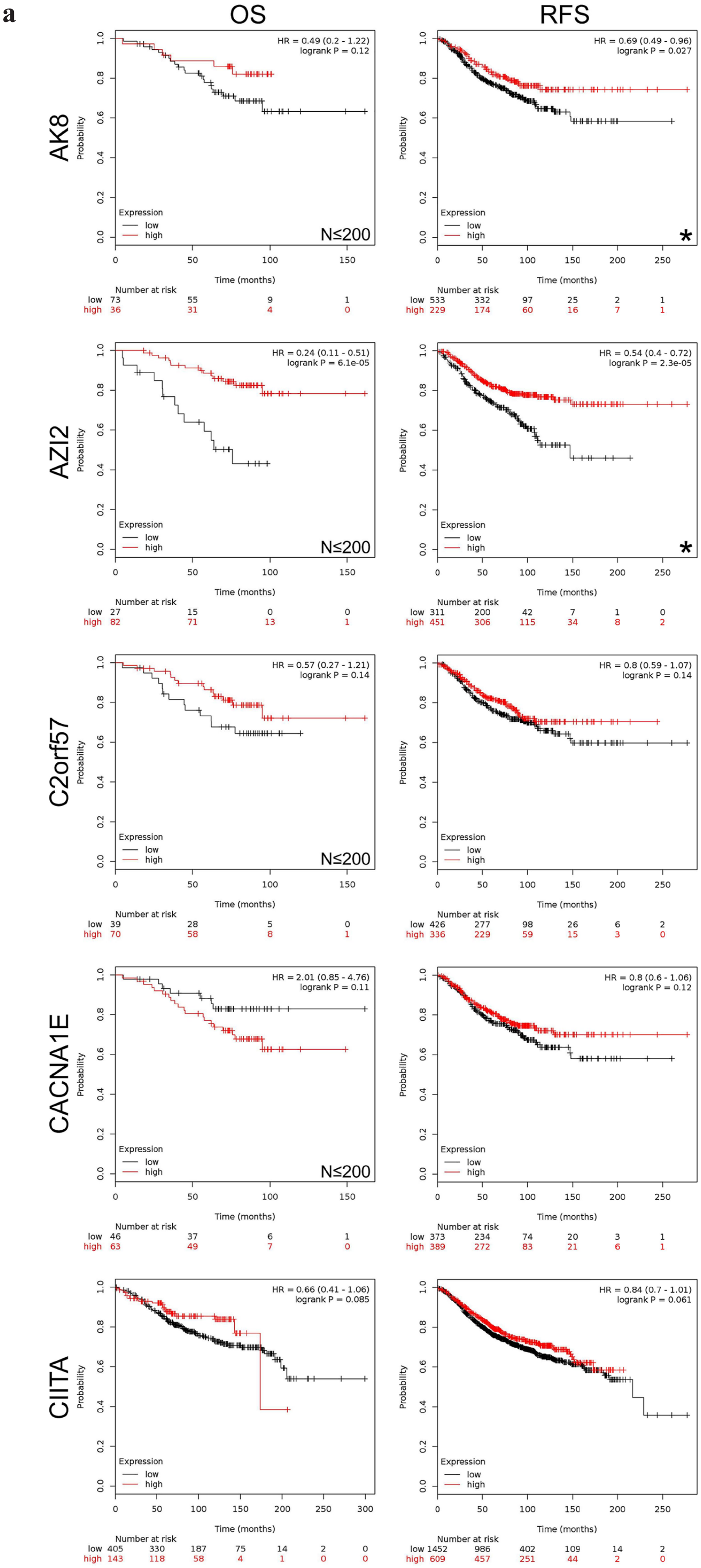

a
OS
RFS
AK8
HR = 0.49 (0.2 - 1.22)
logrank P = 0.12
N≤200
Number at risk
low 73 55 9 1
high 36 31 4 0
HR = 0.69 (0.49 - 0.96)
logrank P = 0.027
*
Number at risk
low 533 332 97 25 2 1
high 229 174 60 16 7 1
AZI2
HR = 0.24 (0.11 - 0.51)
logrank P = 6.1e-05
N≤200
Number at risk
low 27 15 0 0
high 82 71 13 1
HR = 0.54 (0.4 - 0.72)
logrank P = 2.3e-05
*
Number at risk
low 311 200 42 7 1 0
high 451 306 115 34 8 2
C2orf57
HR = 0.57 (0.27 - 1.21)
logrank P = 0.14
N≤200
Number at risk
low 39 28 5 0
high 70 58 8 1
HR = 0.8 (0.59 - 1.07)
logrank P = 0.14
Number at risk
low 426 277 98 26 6 2
high 336 229 59 15 3 0
CACNA1E
HR = 2.01 (0.85 - 4.76)
logrank P = 0.11
N≤200
Number at risk
low 46 37 6 1
high 63 49 7 0
HR = 0.8 (0.6 - 1.06)
logrank P = 0.12
Number at risk
low 373 234 74 20 3 1
high 389 272 83 21 6 1
CIITA
HR = 0.66 (0.41 - 1.06)
logrank P = 0.085
Number at risk
low 405 330 187 75 14 2 0
high 143 118 58 4 1 0 0
HR = 0.84 (0.7 - 1.01)
logrank P = 0.061
Number at risk
low 1452 986 402 109 14 2
high 609 457 251 44 2 0
Probability
Time (months)
Expression
low
high

(*Continued*)

b

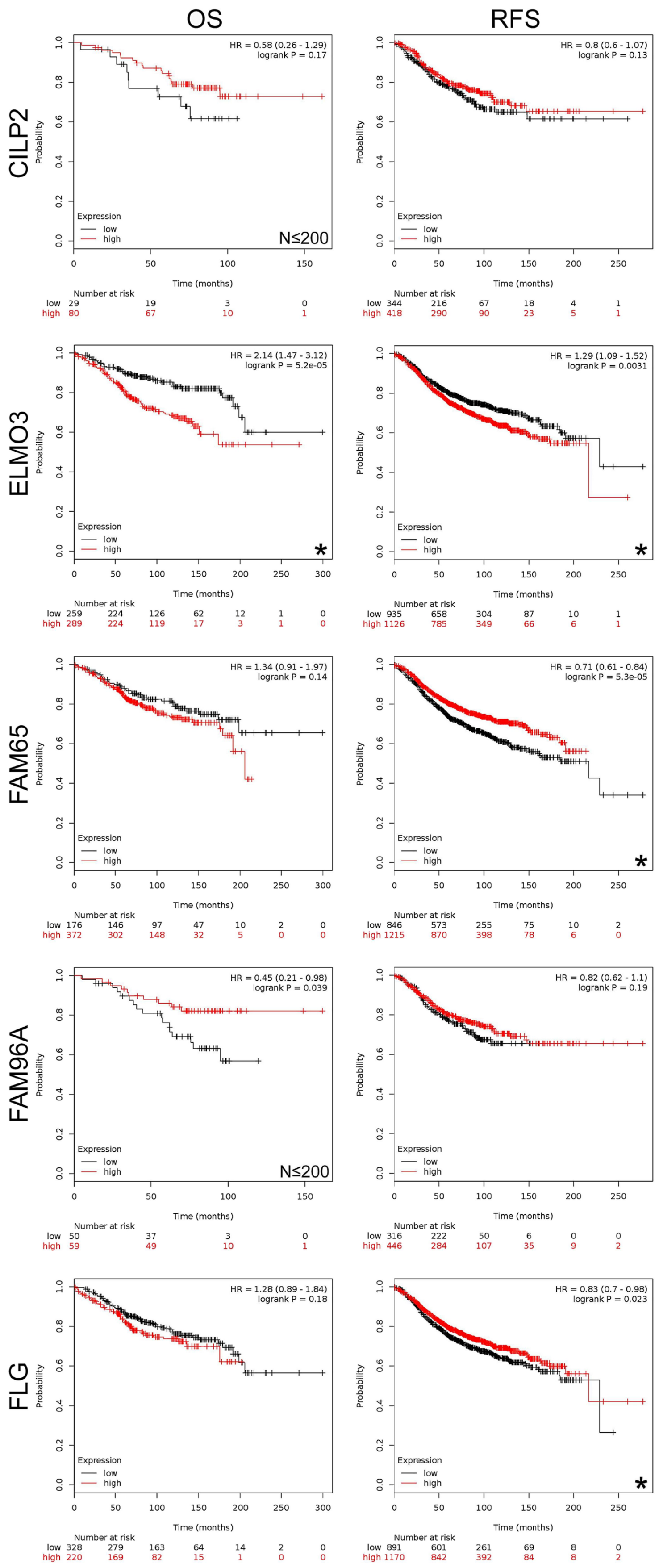
OS
RFS
CILP2
HR = 0.58 (0.26 - 1.29)
logrank P = 0.17
N≤200
Number at risk
low 29 19 3 0
high 80 67 10 1
HR = 0.8 (0.6 - 1.07)
logrank P = 0.13
Number at risk
low 344 216 67 18 4 1
high 418 290 90 23 5 1
ELMO3
HR = 2.14 (1.47 - 3.12)
logrank P = 5.2e-05
*
Number at risk
low 259 224 126 62 12 1 0
high 289 224 119 17 3 1 0
HR = 1.29 (1.09 - 1.52)
logrank P = 0.0031
*
Number at risk
low 935 658 304 87 10 1
high 1126 785 349 66 6 1
FAM65
HR = 1.34 (0.91 - 1.97)
logrank P = 0.14
Number at risk
low 176 146 97 47 10 2 0
high 372 302 148 32 5 0 0
HR = 0.71 (0.61 - 0.84)
logrank P = 5.3e-05
*
Number at risk
low 846 573 255 75 10 2
high 1215 870 398 78 6 0
FAM96A
HR = 0.45 (0.21 - 0.98)
logrank P = 0.039
N≤200
Number at risk
low 50 37 3 0
high 59 49 10 1
HR = 0.82 (0.62 - 1.1)
logrank P = 0.19
Number at risk
low 316 222 50 6 0 0
high 446 284 107 35 9 2
FLG
HR = 1.28 (0.89 - 1.84)
logrank P = 0.18
Number at risk
low 328 279 163 64 14 2 0
high 220 169 82 15 1 0 0
HR = 0.83 (0.7 - 0.98)
logrank P = 0.023
*
Number at risk
low 891 601 261 69 8 0
high 1170 842 392 84 8 2
Probability
Time (months)
Expression
low
high

(*Continued*)

c

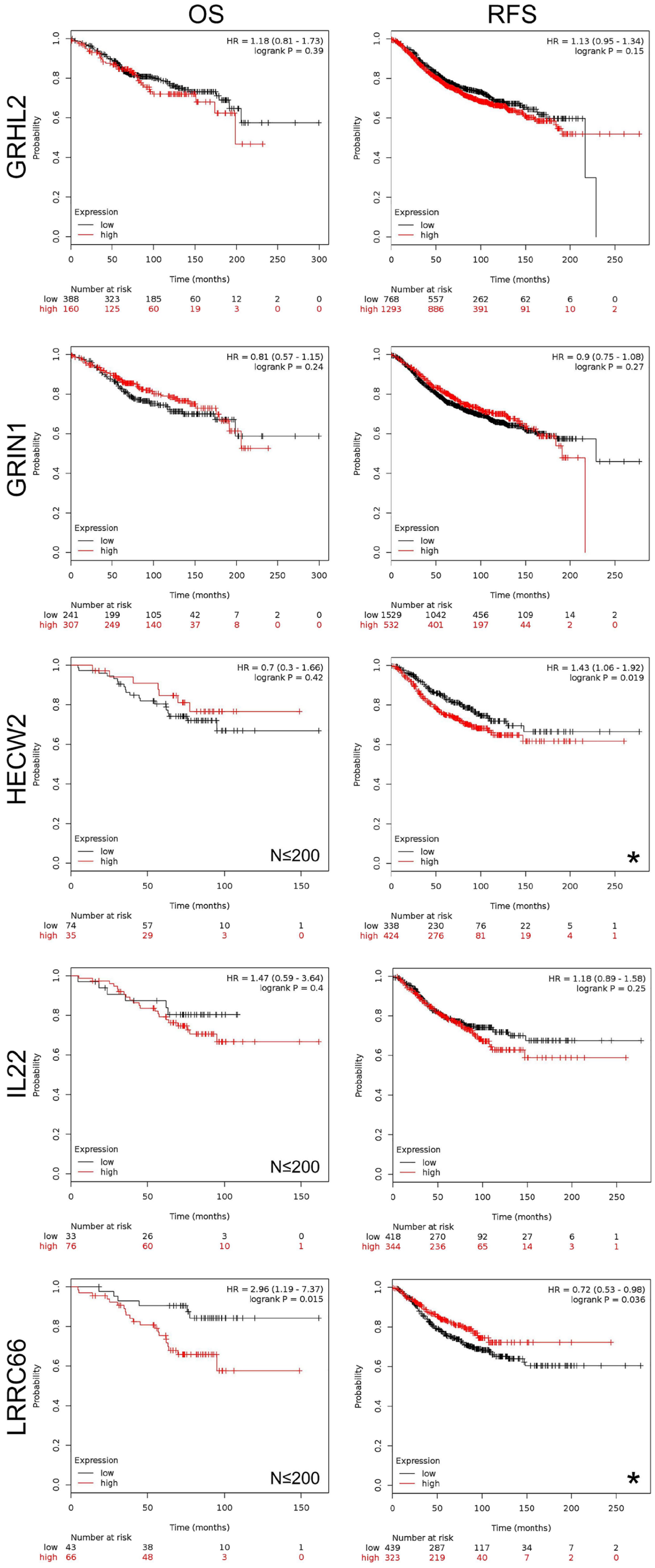
OS
RFS
GRHL2
HR = 1.18 (0.81 - 1.73)
logrank P = 0.39
HR = 1.13 (0.95 - 1.34)
logrank P = 0.15
Number at risk
low 388 323 185 60 12 2 0
high 160 125 60 19 3 0 0
low 768 557 262 62 6 0
high 1293 886 391 91 10 2
GRIN1
HR = 0.81 (0.57 - 1.15)
logrank P = 0.24
HR = 0.9 (0.75 - 1.08)
logrank P = 0.27
low 241 199 105 42 7 2 0
high 307 249 140 37 8 0 0
low 1529 1042 456 109 14 2
high 532 401 197 44 2 0
HECW2
HR = 0.7 (0.3 - 1.66)
logrank P = 0.42
N≤200
HR = 1.43 (1.06 - 1.92)
logrank P = 0.019
*
low 74 57 10 1
high 35 29 3 0
low 338 230 76 22 5 1
high 424 276 81 19 4 1
IL22
HR = 1.47 (0.59 - 3.64)
logrank P = 0.4
N≤200
HR = 1.18 (0.89 - 1.58)
logrank P = 0.25
low 33 26 3 0
high 76 60 10 1
low 418 270 92 27 6 1
high 344 236 65 14 3 1
LRRC66
HR = 2.96 (1.19 - 7.37)
logrank P = 0.015
N≤200
HR = 0.72 (0.53 - 0.98)
logrank P = 0.036
*
low 43 38 10 1
high 66 48 3 0
low 439 287 117 34 7 2
high 323 219 40 7 2 0
Expression
low
high
Probability
Time (months)

(*Continued*)

d

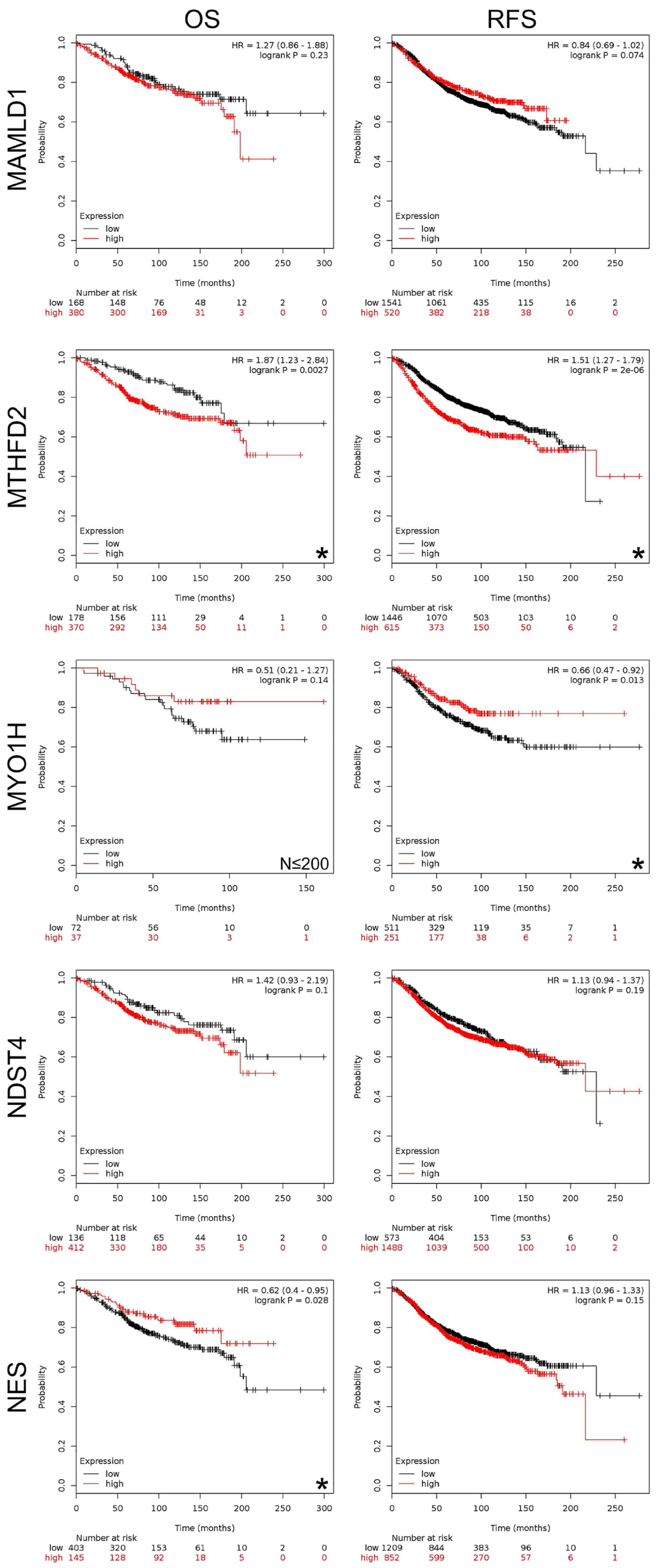

OS
RFS
MAMLD1
HR = 1.27 (0.86 - 1.88)
logrank P = 0.23
HR = 0.84 (0.69 - 1.02)
logrank P = 0.074
MTHFD2
HR = 1.87 (1.23 - 2.84)
logrank P = 0.0027
HR = 1.51 (1.27 - 1.79)
logrank P = 2e-06
MYO1H
HR = 0.51 (0.21 - 1.27)
logrank P = 0.14
N≤200
HR = 0.66 (0.47 - 0.92)
logrank P = 0.013
NDST4
HR = 1.42 (0.93 - 2.19)
logrank P = 0.1
HR = 1.13 (0.94 - 1.37)
logrank P = 0.19
NES
HR = 0.62 (0.4 - 0.95)
logrank P = 0.028
HR = 1.13 (0.96 - 1.33)
logrank P = 0.15
Probability
Time (months)
Expression
low
high
Number at risk

(*Continued*)

e

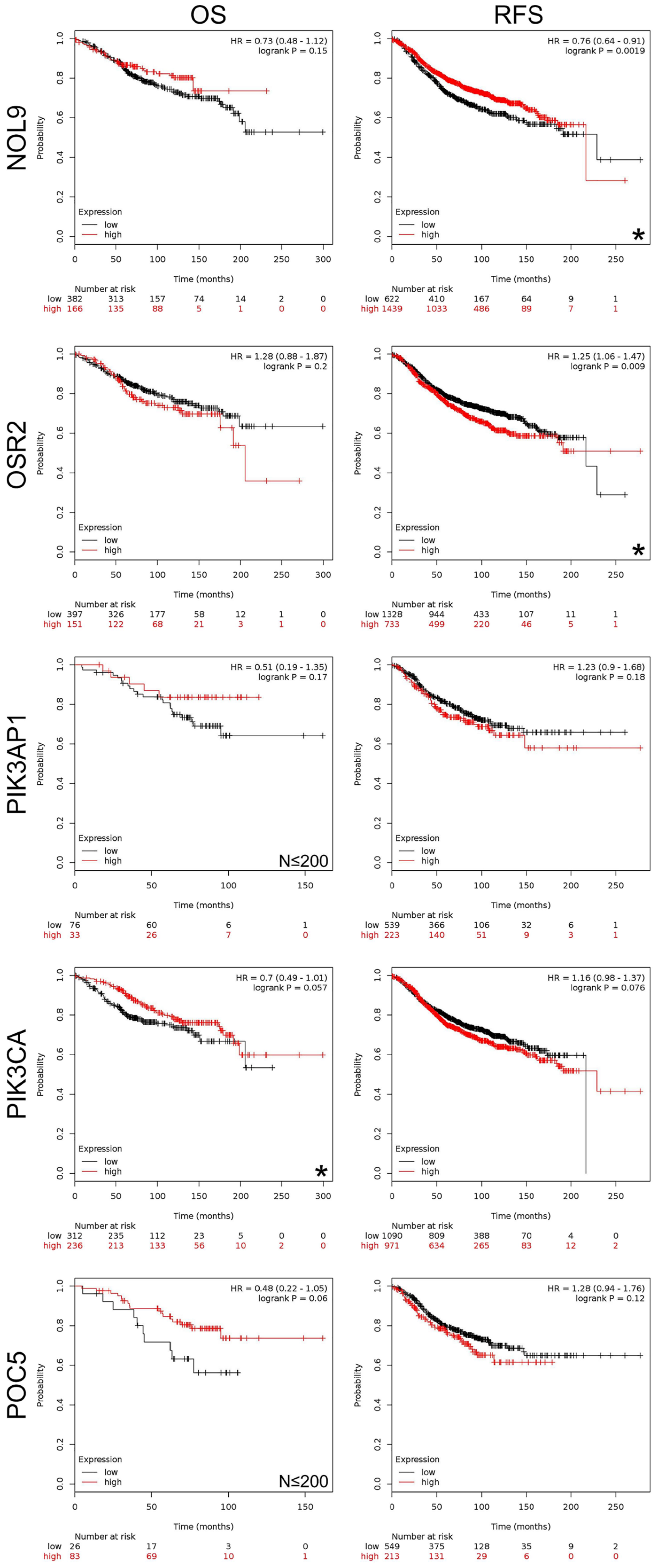

OS
RFS
NOL9
HR = 0.73 (0.48 - 1.12)
logrank P = 0.15
HR = 0.76 (0.64 - 0.91)
logrank P = 0.0019
Probability
Time (months)
Expression
low
high
*
Number at risk
low 382 313 157 74 14 2 0
high 166 135 88 5 1 0 0
low 622 410 167 64 9 1
high 1439 1033 486 89 7 1
OSR2
HR = 1.28 (0.88 - 1.87)
logrank P = 0.2
HR = 1.25 (1.06 - 1.47)
logrank P = 0.009
low 397 326 177 58 12 1 0
high 151 122 68 21 3 1 0
low 1328 944 433 107 11 1
high 733 499 220 46 5 1
PIK3AP1
HR = 0.51 (0.19 - 1.35)
logrank P = 0.17
N≤200
HR = 1.23 (0.9 - 1.68)
logrank P = 0.18
low 76 60 6 1
high 33 26 7 0
low 539 366 106 32 6 1
high 223 140 51 9 3 1
PIK3CA
HR = 0.7 (0.49 - 1.01)
logrank P = 0.057
HR = 1.16 (0.98 - 1.37)
logrank P = 0.076
low 312 235 112 23 5 0 0
high 236 213 133 56 10 2 0
low 1090 809 388 70 4 0
high 971 634 265 83 12 2
POC5
HR = 0.48 (0.22 - 1.05)
logrank P = 0.06
N≤200
HR = 1.28 (0.94 - 1.76)
logrank P = 0.12
low 26 17 3 0
high 83 69 10 1
low 549 375 128 35 9 2
high 213 131 29 6 0 0

(*Continued*)

f

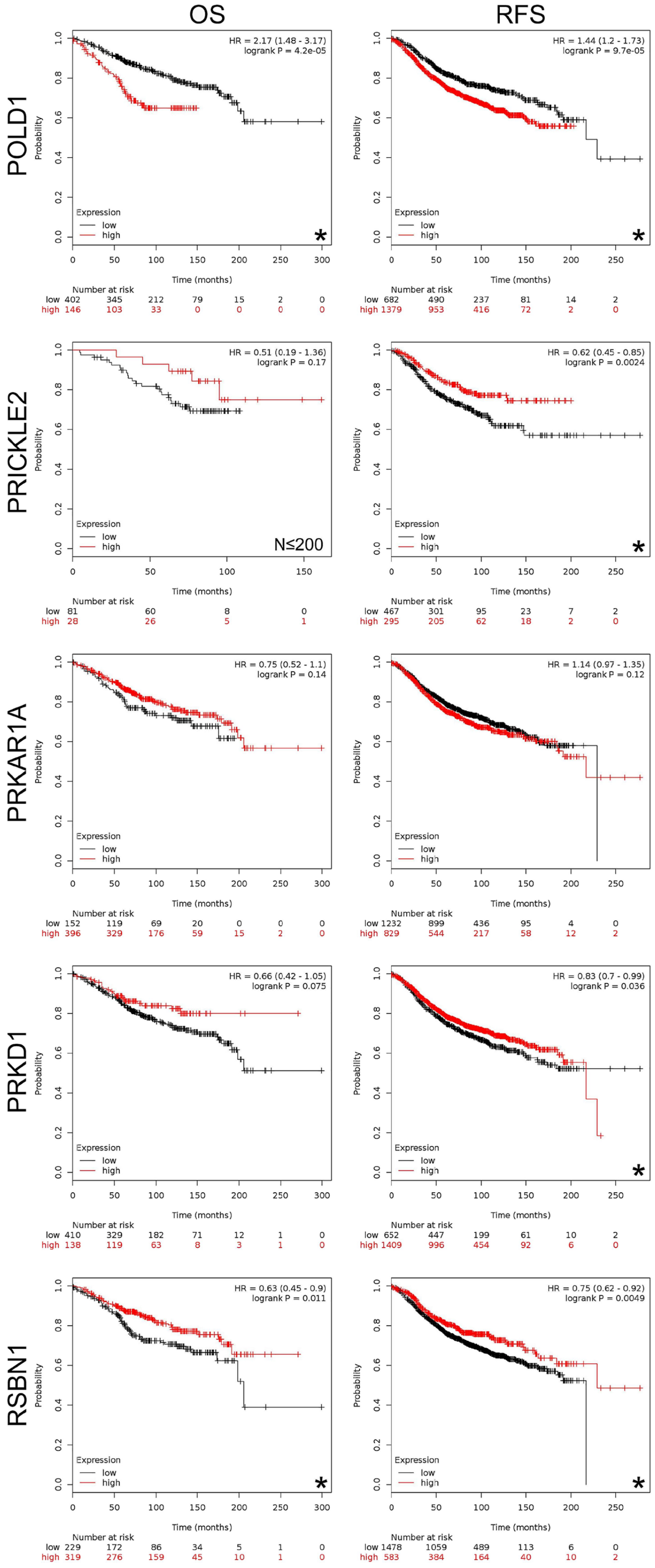
OS
RFS
POLD1
PRICKLE2
PRKAR1A
PRKD1
RSBN1
HR = 2.17 (1.48 - 3.17) logrank P = 4.2e-05
HR = 1.44 (1.2 - 1.73) logrank P = 9.7e-05
HR = 0.51 (0.19 - 1.36) logrank P = 0.17
N≤200
HR = 0.62 (0.45 - 0.85) logrank P = 0.0024
HR = 0.75 (0.52 - 1.1) logrank P = 0.14
HR = 1.14 (0.97 - 1.35) logrank P = 0.12
HR = 0.66 (0.42 - 1.05) logrank P = 0.075
HR = 0.83 (0.7 - 0.99) logrank P = 0.036
HR = 0.63 (0.45 - 0.9) logrank P = 0.011
HR = 0.75 (0.62 - 0.92) logrank P = 0.0049
Expression
low
high
Probability
Time (months)
Number at risk

(*Continued*)

g

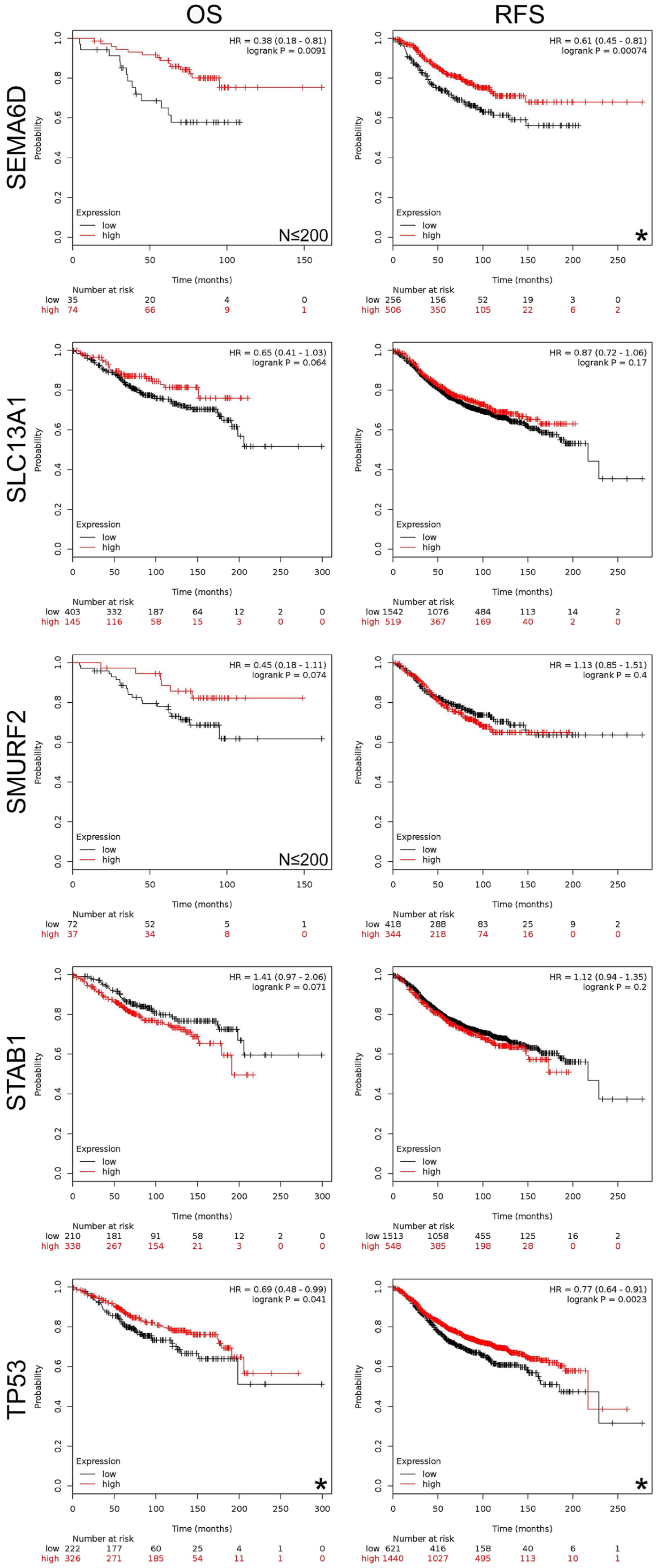

OS
RFS
SEMA6D
HR = 0.38 (0.18 - 0.81)
logrank P = 0.0091
N≤200
HR = 0.61 (0.45 - 0.81)
logrank P = 0.00074
*
Number at risk
low 35 20 4 0
high 74 66 9 1
low 256 156 52 19 3 0
high 506 350 105 22 6 2
SLC13A1
HR = 0.65 (0.41 - 1.03)
logrank P = 0.064
HR = 0.87 (0.72 - 1.06)
logrank P = 0.17
low 403 332 187 64 12 2 0
high 145 116 58 15 3 0 0
low 1542 1076 484 113 14 2
high 519 367 169 40 2 0
SMURF2
HR = 0.45 (0.18 - 1.11)
logrank P = 0.074
N≤200
HR = 1.13 (0.85 - 1.51)
logrank P = 0.4
low 72 52 5 1
high 37 34 8 0
low 418 288 83 25 9 2
high 344 218 74 16 0 0
STAB1
HR = 1.41 (0.97 - 2.06)
logrank P = 0.071
HR = 1.12 (0.94 - 1.35)
logrank P = 0.2
low 210 181 91 58 12 2 0
high 338 267 154 21 3 0 0
low 1513 1058 455 125 16 2
high 548 385 198 28 0 0
TP53
HR = 0.69 (0.48 - 0.99)
logrank P = 0.041
*
HR = 0.77 (0.64 - 0.91)
logrank P = 0.0023
*
low 222 177 60 25 4 1 0
high 326 271 185 54 11 1 0
low 621 416 158 40 6 1
high 1440 1027 495 113 10 1
Probability
Time (months)
Expression
low
high

(Continued)

h

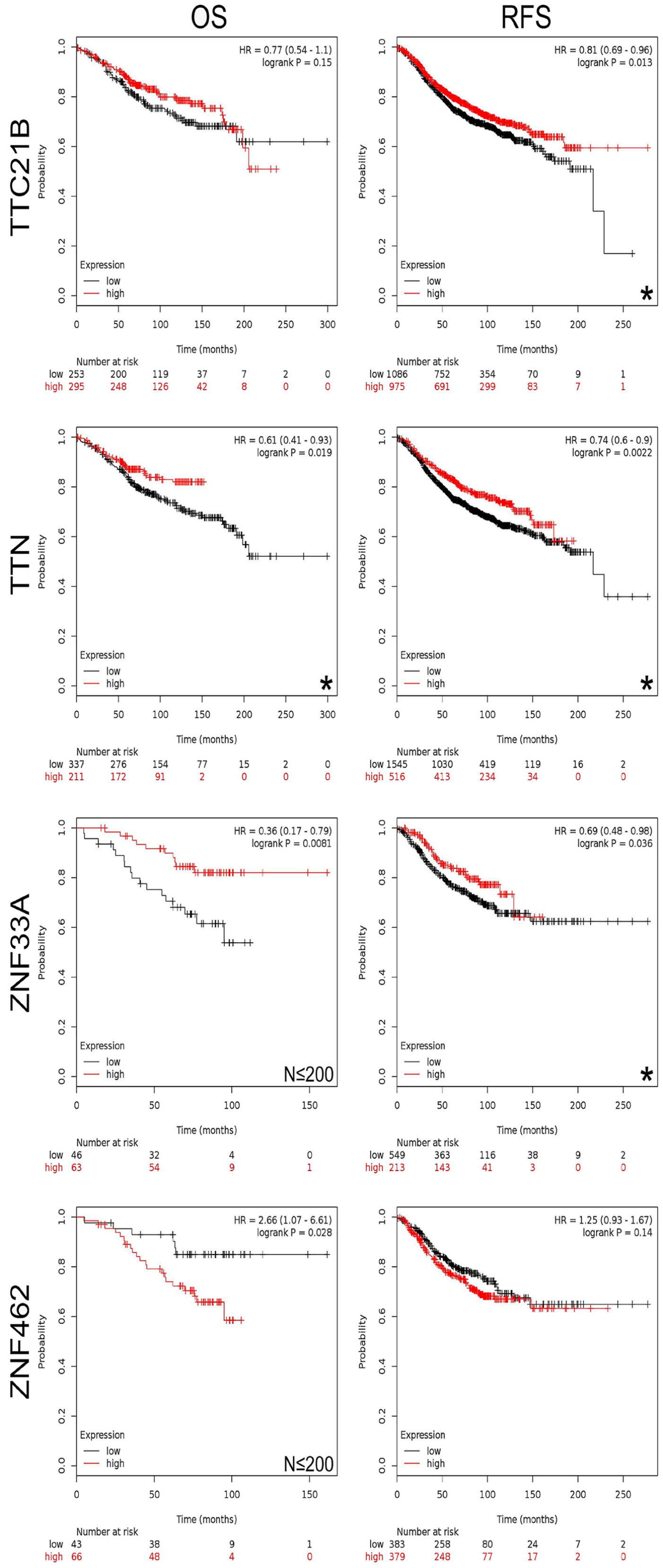

Supplementary Figure 1: (a-h) Overall survival and relapse free survival. The "Kaplan-Meier Plotter" (KM Plotter) database was used to verify the overall survival (OS) and relapse free survival (RFS) of luminal breast cancer patients (independent of HER-2 status) considering high and low expression of gene candidates. The following parameters have been set: the best performing threshold was used as the cutoff and the analysis was restricted by filtering "ER positive" samples in the ER status. It was not possible to verify PLA2G4D gene data, since probes in the database were not available. ≤200: number of samples available were between 50 and 200; *: p≤0.05.

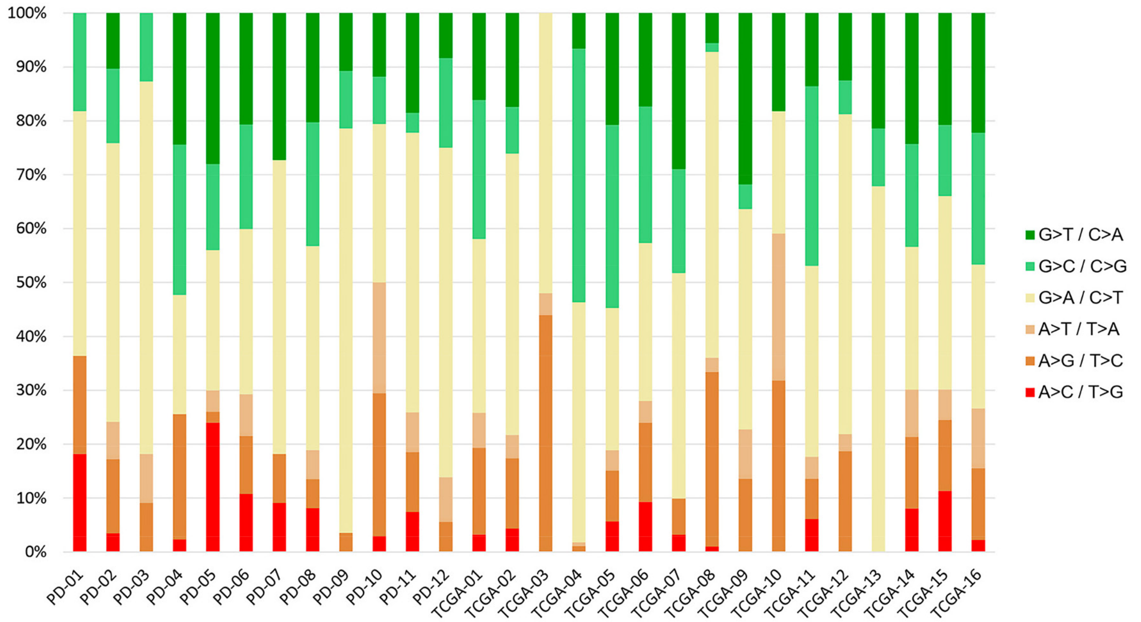

Supplementary Figure 2: Most frequent base substitutions present in the 29 tumor samples previously reported in COSMIC literature, including synonymous variants.

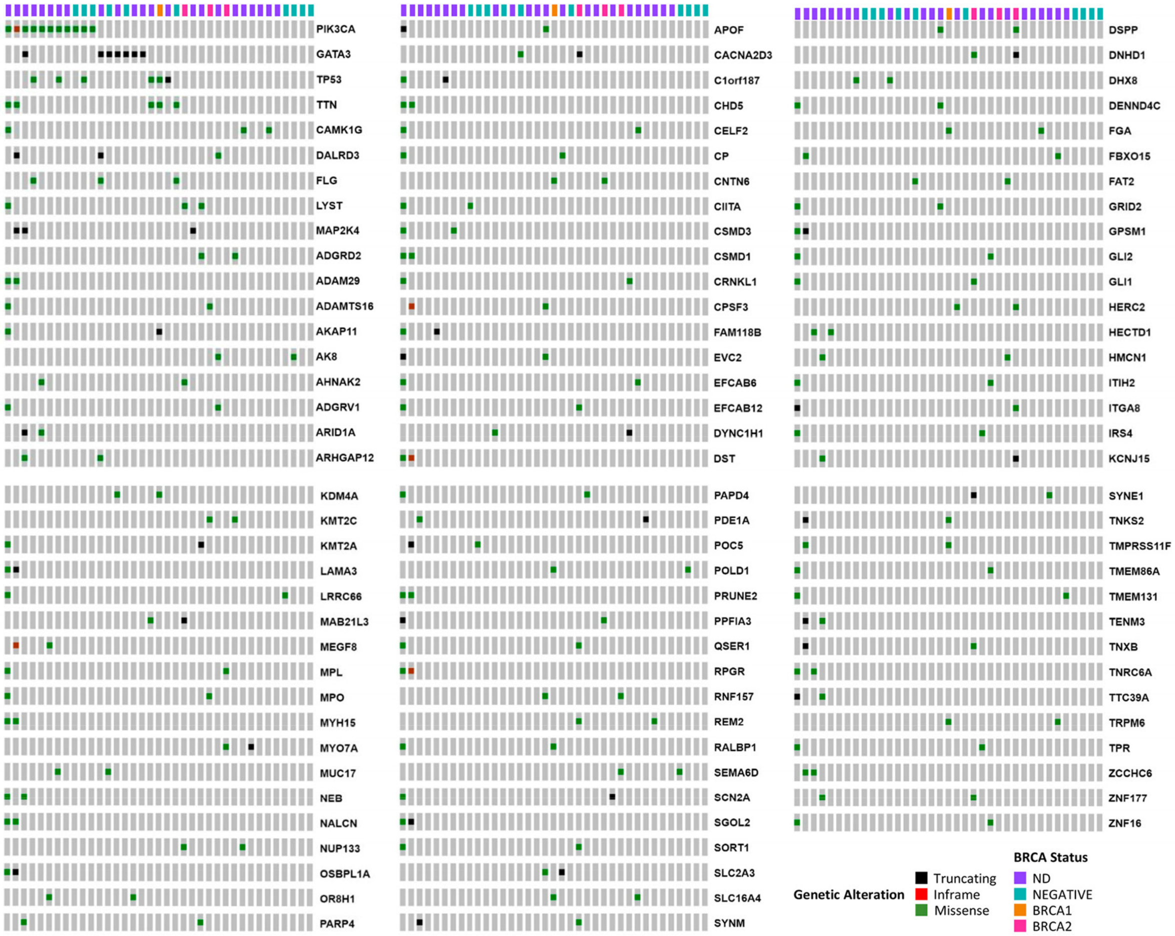

Supplementary Figure 3: Most frequently mutated genes in 37 luminal tumors from young patients (current study + COSMIC database + literature). The figure was obtained using OncoPrinter, a cBioPortal tool (Cerami E et.al.; 2012). The setup used excluded synonymous mutations, and included just genes with 2 or more SNV's in different patients. After getting the "raw" figure, it was edited adding *BRCA 1* and *BRCA 2* status. Only two samples from the current study did not present concomitant SNV's in the same genes.

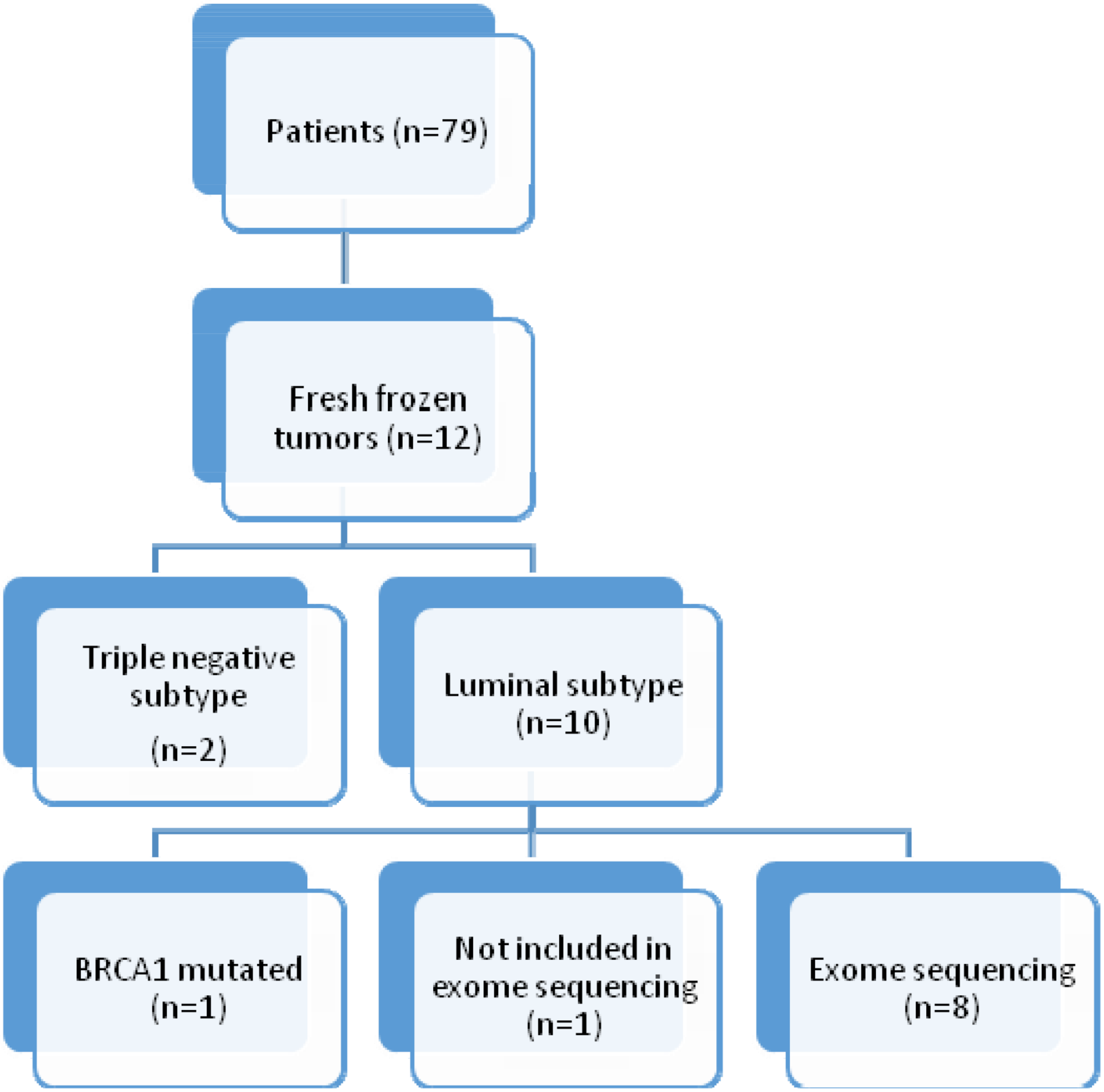

Supplementary Figure 4: Number of patients included and number tumor samples collected and analyzed in the current study.

Supplementary Table 1: Clinical and pathological characteristics, BRCA sequencing and MLPA results

ID: identification of patients; HT, histological tumor; IDC: invasive ductal carcinoma; ILC: invasive lobular carcinoma; HG, histological grade; CS, clinical stage; FH, family history; Lim, limited; ND: not determined; mut, mutation; wt, wild type; MLPA, Multiplex Ligation-Dependent Probe Amplification.

See Supplementary File 1

Supplementary Table 2: *BRCA1* gene variants

NT, nucleotide; HGVS, Human Genome Variation Society; Syn, Synonymous; F, Frameshift; IVS, Intervening Sequence; M, Missense; IFD, In Frame Deletion; ND, not determined; MAF, minor allele frequency; BIC, Breast Cancer Information Core; LOVD, Leiden Open Variation Database; LOVD-IARC, Leiden Open Variation Database - International Agency for Research on Cancer; UMD, Mutation Database Universal; UV, unclassified variant; Uncertain Significance , Variant of Uncertain Significance; n, number of patients harboring the Single Nucleotide Variant (SNV).

See Supplementary File 1

Supplementary Table 3: *BRCA2* gene variants

NT, nucleotide; HGVS, Human Genome Variation Society; Syn, Synonymous; F, Frameshift; IVS, Intervening Sequence; M, Missense; ND, not determined; MAF, minor allele frequency; BIC, Breast Cancer Information Core; LOVD, Leiden Open Variation Database; LOVD-IARC, Leiden Open Variation Database - International Agency for Research on Cancer; UMD, Mutation Database Universal; UV, unclassified variant; Uncertain Significance, Variant of Uncertain Significance; n, number of patients with the variant.

See Supplementary File 1

Supplementary Table 4: *In silico* analysis of VUS (missense and in frame deletions) identified in *BRCA1* and *BRCA2* genes, using mutation function prediction models

HGVS, Human Genome Variation Society; HGMD, Human Gene Mutation Database; PolyPhen, Polymorphism Phenotyping; SIFT, Sorting Intolerant From Tolerant; Provean, Protein Variation Effect Analyzer; ESE, Exonic Splicing Enhancers; ESS, Exonic Splicing Silencers; ID, identification of two patients presenting more than one VUS (A or B). Positive results are highlighted (bold). Align GVGD: Class C0, C15, C25, C35 (Less likely to interfere in protein function), C45, C55, C65 (More likely to interfere in protein function).

See Supplementary File 1

Supplementary Table 5: Sequencing depths of tumor and normal samples

Sample	ID	Average	Stdev
Tumor	402	29.2838	24.5808
	413	35.2194	27.5934
	406	36.5737	29.7472
	416	37.6557	31.5998
	401	37.4398	30.2831
	404	38.9684	31.2011
	415	29.5658	26.5644
	417	41.6143	32.7913
	**Mean**	35.7901	29.2951
Normal	402	41.629	32.3451
	413	31.8173	24.804
	406	20.9754	17.2859
	416	44.3717	33.9972
	401	36.545	28.6825
	404	38.3545	30.0327
	415	34.2078	26.1965
	417	42.46	32.3717
	**Mean**	36.2951	28.2145

ID, identification of patients.

Supplementary Table 6: Mutation Rates per Megabase observed in tumors

Function:	NA	nonsynonymous	stopgain	synonymous	unknown	TOTAL	synonymous
**Min.**	1.0000	0.1351	0.0000	0.0811	0.0000	1.3240	1.2160
**1st Qu.**	1.1490	0.2095	0.0000	0.0811	0.0000	1.6350	1.5540
**Median**	1.4050	0.2838	0.0135	0.0946	0.0000	1.8240	1.6760
**Mean**	1.4830	0.2838	0.0135	0.1520	0.0068	1.9390	1.7870
**3rd Qu.**	1.6080	0.3311	0.0270	0.1554	0.0068	2.1150	1.9460
**Max.**	2.5410	0.4865	0.0270	0.4324	0.0270	3.0540	2.9190

synonymous means the mutation rates were calculated for all SNVs whose function was labeled as anything except synonymous.

Supplementary Table 7: Predicted effects for single nucleotide variants observed in exome sequencing

Predicted effects	ID								Total
	402	413	406	416	401	404	415	417	
3_prime_UTR_ variant	14	4	4	9	4	7	8	8	**58**
5_prime_UTR_ premature_start_ codon_gain_variant	0	2	0	0	0	0	0	0	**2**
5_prime_UTR_ variant	2	1	0	0	0	1	1	0	**5**
downstream_gene_ variant	3	2	0	3	1	3	3	3	**18**
intergenic_region	21	9	2	9	3	7	5	8	**64**
intron_variant	15	9	3	6	1	2	5	4	**45**
missense _variant	10	7	7	6	6	4	5	5	**50**
non_coding_exon_ variant	3	1	1	3	3	0	5	3	**19**
splice_region_variant	0	3	1	2	1	2	1	0	**10**
splice_donor_variant	0	1	0	0	0	1	0	0	**2**
stop_gained	0	0	0	1	0	1	1	1	**4**
synonymous_variant	4	0	1	1	3	3	11	1	**24**
upstream_gene_ variant	2	1	0	2	1	0	1	2	**9**
**Total**	**74**	**40**	**19**	**42**	**23**	**31**	**46**	**35**	**310**

ID, identification of patients.

Supplementary Table 8: Confirmation of SNVs by capillary sequencing. ID, identification of patient

See Supplementary File 1

Supplementary Table 8a: Oligonucleotides for gene sequencing

See Supplementary File 1

Supplementary Table 9: Score system used in Table 1

A score system was used to identify potential cancer drivers: a) genes found in Cancer Genes Census, CGC, database: 3 points; b) known/functional mutation domain; frequency of the variant in other cancers and/or in breast cancer (>1%): 0.5 points each; c) mutation consequence when nonsense was scored 1.5 points; d) KM-OS (overall survival) p-value using the KM Plot Database (http://kmplot.com/analysis/): 1 point (if $p \leq 0.05$ and $n \geq 200$); literature association with breast cancer or other cancer; mutation function assessment algorithms, FATHMM (http://fathmm.biocompute.org.uk/; v.2.3) using MKL algorithm (predictions on both coding and non-coding variants, pathogenic or neutral), PolyPhen-2 (http://genetics.bwh.harvard.edu/pph2/; v.2; deleterious or benign), SIFT (http://sift.jcvi.org/; tolerated or not tolerated), AlignGV-GD (http://agvgd.hci.utah.edu/); Class C0, C15, C25, C35, (Less likely to interfere in protein function) C45, C55, C65 (More likely to interfere in protein function) and CRAVAT (https://www.cravat.us/CRAVAT/; v.4.3; $p \leq 0.05$): 1 point, if the variant was considered pathogenic at least in 3 of them; e) CCGD was scored according to the rank: “A”: 2 points, “B”: 1.5 points, “C”: 1 point, “D”: 0.5 point. “Not Ranked”: NR variants were not scored. SNVs Frequency in all cancers: SNVs (including synonymous) in all types of cancers. Classification of gene variants: PD: gene variants considered probably driver, attaining score ≥3.5; pd: gene variants considered possibly drivers, scored 2-3; Neutral: gene variants scoring ≤1.5.

See Supplementary File 1

Supplementary Table 9a: Table 1 references

See Supplementary File 1

Supplementary Table 10: Potential cancer driver genes in luminal breast cancer (HER2 negative), from 29 young patients identified in COSMIC database and Banerji *et.al* (2012) study (blue color indicates that the gene is mutated in another patient(s))

See Supplementary File 1

Supplementary Table 10a. Scoring system used in Supplementary Table 10

Each non-synonymous SNV was scored according to the following criteria: a) genes found in cancer gene census, CGC: 3 points; b) genes listed in the Candidate Cancer Gene Database, CCGD were scored according to the rank: “A”: 2 points, “B”: 1.5 points, “C”: 1 point, “D”: 0.5 points. “Not Ranked”: NR variants were not scored; c) Nonsense and Frameshift: 1.5; In-frame, Intronic (+/-3) nucleotides: 1 point; d) known/functional mutation domain or frequency of the variant in other cancers and/or in breast cancer (≥1%): 0.5 point each; e) mutation function assessment algorithms, FATHMM (http://fathmm.biocompute.org.uk/; v.2.3) using MKL algorithm (predictions on both coding and non-coding variants, pathogenic or neutral), PolyPhen-2 (http://genetics.bwh.harvard.edu/pph2/; v.2; deleterious or benign), SIFT(http://sift.jcvi.org/; tolerated or not tolerated): 1 point, if the variant was considered pathogenic in at least 2 of them; e) CRAVAT (https://www.cravat.us/CRAVAT/; v.4.3; $p \leq 0.05$): 1 point.

See Supplementary File 1

Supplementary Table 11: Biological function (via GO category or Interpro process), using gene variants in luminal tumors from 29 young patients, identified in the literature and/or the COSMIC database. DAVID (Database for Annotation, Visualization and Integrated Discovery. Huang DW, Sherman BT, Lempicki RA. Systematic and integrative analysis of large gene lists using DAVID bioinformatics resources. Nat Protoc [Internet]. 2008; 4: 44–57. https://doi.org/10.1038/nprot.2008.211) was used to identify enriched biological functions. If more than one GO category was identified ($p \leq 0.05$), the one consisting of the largest number of genes was chosen.

See Supplementary File 1

Supplementary Table 12: Number of potential driver genes per tumor sample, according to the score system (1 or 2). Genes presented in Supplementary Table 10 , which are classified as cancer genes census in the CGC database (http://cancer.sanger.ac.uk/cosmic/census) or candidate cancer driver genes, ranked as A or B in the “Candidate cancer gene database” (http://ccgd-starrlab.oit.umn.edu/about.php) per patient. All 37 tumor samples, 8 current analyses and 29 from literature or COSMIC database were included in the analysis. Data about the number of nonsense, frameshift and other types of mutations, pd and PD per patient and genes that have role in positive regulation of gene expression are also shown

See Supplementary File 1

Supplementary Table 13: Oligonucleotides for *BRCA1* gene sequencing. F, forward; R, reverse

See Supplementary File 1

Supplementary Table 14: Oligonucleotides for *BRCA2* gene sequencing. F, forward; R, reverse

See Supplementary File 1

Supplementary Table 15: Single nucleotide variants SNVs detected in the exome sequencing. GT, Genotype; IGT, genotype when called independently (only filled if called in joint prior mode); DP, total read depth; DP4, high-quality ref-forward bases, ref-reverse, alt-forward and alt-reverse bases; BCOUNT, occurrence count for each base at this site (A, C, G, T); GQ, genotype quality; JGQ, joint genotype quality (only filled if called in join prior mode); VAQ, variant allele quality; BQ, average base quality; MQ, average mapping quality across all reads; AMQ, average mapping quality for each allele present in the genotype; SS, variant status relative to non-adjacent Normal, 0=wild type, 1=germline, 2=somatic, 3=LOH, 4=unknown; SSC, somatic score

See Supplementary File 1
